# Supplementary material for: Anticoagulants utilization in eight hospitals within the Luzhou region from 2019 to 2023
Source: PLoS One. 2025 Jan 31;20(1):e0318463. doi: 10.1371/journal.pone.0318463 (PMC11785323; doi:10.1371/journal.pone.0318463)
Supplement: S1 Table — (DOCX) [file pone.0318463.s001.docx]

Supplementary Table 1: Annual trends in the utilization of four anticoagulants at the eight hospitals during the study period.

|  | Annual consumption of anticoagulant drug (DDDs) | | | | | p | Trend |
| --- | --- | --- | --- | --- | --- | --- | --- |
|  | 2019 | 2020 | 2021 | 2022 | 2023 |  |  |
| Warfarin | | | | | | | |
| Hospital A | 279277 | 264703 | 286315 | 198825 | 207169 | 0.100 | - |
| Hospital B | 41343 | 36856 | 37792 | 26660 | 21301 | 0.016 | Decreasing |
| Hospital C | 6234 | 7769 | 9378 | 10155 | 10845 | 0.003 | Increasing |
| Hospital D | 13652 | 22118 | 23687 | 15689 | 14461 | 0.792 | - |
| Hospital E | 14910 | 19246 | 20403 | 16958 | 14850 | 0.808 | - |
| Hospital F | 4174 | 4423 | 6010 | 6023 | 6507 | 0.017 | Increasing |
| Hospital G | 780 | 3180 | 4460 | 3900 | 2120 | 0.544 | - |
| Hospital H | 7890 | 10737 | 13243 | 11368 | 11618 | 0.231 | - |
| Rivaroxaban | | | | | | | |
| Hospital A | 93443 | 95563 | 128232 | 236272 | 358435 | 0.023 | Increasing |
| Hospital B | 13658 | 19945 | 29474 | 84848 | 122667 | 0.017 | Increasing |
| Hospital C |  | 353 | 5333 | 24938 | 40870 | 0.021 | Increasing |
| Hospital D |  |  | 8940 | 55954 | 102724 | 0.029 | Increasing |
| Hospital E | 1241 | 3260 | 7703 | 40134 | 62264 | 0.023 | Increasing |
| Hospital F |  |  |  | 294 | 2982 | 0.138 | - |
| Hospital G |  |  |  | 700 | 1547 | 0.051 | - |
| Hospital H |  |  | 662 | 7131 | 13795 | 0.037 | Increasing |
| Heparin sodium | | | | | | | |
| Hospital A | 22959 | 21060 | 18124 | 12839 | 11429 | 0.003 | Decreasing |
| Hospital B | 35709 | 12730 | 5905 | 8829 | 13168 | 0.229 | - |
| Hospital C | 9771 | 15849 | 21780 | 17240 | 17261 | 0.286 | - |
| Hospital D | 1119 | 2134 | 2764 | 3248 | 3485 | 0.006 | Increasing |
| Hospital E | 10143 | 10154 | 9976 | 9219 | 9844 | 0.254 | - |
| Hospital F | 1051 | 483 | 2744 | 3708 | 5975 | 0.017 | Increasing |
| Hospital G | 5768 | 5799 | 5025 | 6010 | 7129 | 0.274 | - |
| Hospital H | 30428 | 26576 | 32479 | 23098 | 28569 | 0.605 | - |
| Low molecular weight heparin | | | | | | | |
| Hospital A | 225537 | 316801 | 323935 | 393025 | 346637 | 0.087 | - |
| Hospital B | 134726 | 111295 | 142028 | 138113 | 195561 | 0.139 | - |
| Hospital C | 43758 | 54033 | 56436 | 72407 | 77683 | 0.004 | Increasing |
| Hospital D | 50232 | 93598 | 100486 | 98695 | 79254 | 0.415 | - |
| Hospital E | 23086 | 33016 | 39552 | 83266 | 99715 | 0.011 | Increasing |
| Hospital F | 7569 | 7312 | 10737 | 22808 | 33114 | 0.022 | Increasing |
| Hospital G | 33897 | 28824 | 31482 | 42435 | 56430 | 0.083 | - |
| Hospital H | 63793 | 56533 | 74714 | 74780 | 155197 | 0.108 | - |

Abbreviations: defined daily doses (DDDs).
