# Supplementary material for: Anticoagulants utilization in eight hospitals within the Luzhou region from 2019 to 2023
Source: PLoS One. 2025 Jan 31;20(1):e0318463. doi: 10.1371/journal.pone.0318463 (PMC11785323; doi:10.1371/journal.pone.0318463)
Supplement: S2 Table — (DOCX) [file pone.0318463.s002.docx]

Supplementary Table 2: Inpatient and outpatient DDDs of different anticoagulants in eight hospitals.

|  | 2019 | | 2020 | | 2021 | | 2022 | | 2023 | |
| --- | --- | --- | --- | --- | --- | --- | --- | --- | --- | --- |
|  | Outpatient | Inpatient | Outpatient | Inpatient | Outpatient | Inpatient | Outpatient | Inpatient | Outpatient | Inpatient |
| Hospital A | | | | | | | | | | |
| Warfarin | 232000 | 47277 | 222900 | 41803 | 245273 | 41042 | 175340 | 23485 | 185580 | 21589 |
| Rivaroxaban | 47963 | 45480 | 48693 | 46871 | 67323 | 60909 | 150330 | 85942 | 237453 | 120982 |
| Apixaban |  |  |  |  | 18 |  |  |  |  |  |
| Edoxaban |  |  |  |  |  |  | 32 | 102 | 3444 | 1902 |
| Dabigatran ester | 37 | 29 | 1305 | 305 | 1558 | 130 | 11257 | 2094 | 10006 | 1046 |
| Heparin sodium | 74 | 22885 | 135 | 20925 | 156 | 17968 | 18 | 12821 | 160 | 11269 |
| Heparin calcium |  |  |  |  |  |  |  | 2355 | 10 | 25398 |
| LMWH | 16886 | 208651 | 31190 | 285611 | 43865 | 280070 | 46324 | 346701 | 46401 | 300236 |
| Fondaparinux sodium |  |  | 31 | 468 | 889 | 3163 | 3198 | 605 | 7290 | 154 |
| Bivarudin |  | 3 |  | 111 |  | 641 |  | 1759 |  | 2183 |
| Argatroban |  | 32 | 1 | 108 |  | 113 |  | 84 |  | 49 |
| Hospital B | | | | | | | | | | |
| Warfarin | 31440 | 9903 | 25980 | 10876 | 27480 | 10312 | 21420 | 5240 | 17780 | 3521 |
| Rivaroxaban | 9943 | 3715 | 13858 | 6088 | 17468 | 12006 | 55950 | 28898 | 82425 | 40242 |
| Apixaban |  |  | 39 | 227 | 1533 | 1078 | 217 | 2065 | 3563 | 5712 |
| Edoxaban |  |  |  |  |  |  |  |  | 189 | 137 |
| Dabigatran ester | 1914 | 528 | 3670 | 1162 | 8800 | 2691 | 6699 | 1056 | 5368 | 374 |
| Heparin sodium | 551 | 35158 | 303 | 12428 | 99 | 5806 | 16 | 8813 | 51 | 13116 |
| LMWH | 611 | 134115 | 1763 | 109532 | 2704 | 139325 | 5108 | 133004 | 23891 | 171670 |
| Hospital C | | | | | | | | | | |
| Warfarin | 3032 | 3202 | 4979 | 2791 | 7253 | 2126 | 8354 | 1802 | 9536 | 1309 |
| Rivaroxaban |  | 15 | 143 | 210 | 2370 | 2963 | 10955 | 13983 | 22863 | 18008 |
| Bivarudin |  |  |  |  |  | 43 |  | 339 |  | 2 |
| Heparin sodium | 85 | 9686 | 16 | 15833 | 15 | 21765 | 21 | 17219 | 133 | 17129 |
| Heparin calcium |  |  |  |  |  |  |  | 91 |  | 1477 |
| LMWH | 85 | 43673 | 524 | 53509 | 574 | 55862 | 2746 | 69661 | 2175 | 75508 |
| Hospital D | | | | | | | | | | |
| Warfarin | 9520 | 4132 | 15560 | 6558 | 17340 | 6347 | 12720 | 2969 | 11980 | 2481 |
| Rivaroxaban |  |  |  |  | 4435 | 4505 | 38505 | 17449 | 67980 | 34744 |
| Heparin sodium | 133 | 986 | 264 | 1870 | 307 | 2457 | 281 | 2967 | 76 | 3409 |
| LMWH |  | 50232 | 16 | 93582 |  | 100486 | 262 | 98432 |  | 79254 |
| Hospital E | | | | | | | | | | |
| Warfarin | 11681 | 3229 | 16279 | 2967 | 17065 | 3337 | 14279 | 2680 | 12440 | 2410 |
| Rivaroxaban | 532 | 709 | 1612 | 1648 | 4099 | 3604 | 21730 | 18404 | 37635 | 24629 |
| Dabigatran ester | 29 |  |  |  | 33 |  | 220 |  | 198 |  |
| Heparin sodium |  | 10138 | 9 | 10145 | 18 | 9959 | 63 | 9156 | 55 | 9789 |
| Heparin calcium | 2 | 3853 | 4 | 2886 |  | 4907 |  | 5929 |  | 5005 |
| LMWH | 147 | 22939 | 65 | 32951 | 63 | 39489 | 16152 | 67115 | 144 | 99571 |
| Hospital F | | | | | | | | | | |
| Warfarin | 3913 | 261 | 3931 | 492 | 5603 | 407 | 5560 | 463 | 6224 | 283 |
| Rivaroxaban |  |  |  |  |  |  | 280 | 14 | 1967 | 1015 |
| Argatroban |  |  |  |  |  |  |  | 2 |  | 8 |
| Heparin sodium | 221 | 830 | 205 | 278 | 224 | 2520 | 323 | 3385 | 379 | 5596 |
| LMWH | 20 | 7549 | 163 | 7149 | 22 | 10715 | 9 | 22799 | 46 | 33068 |
| Hospital G | | | | | | | | | | |
| Warfarin | 200 | 580 | 880 | 2300 | 1860 | 2600 | 2000 | 1900 | 1220 | 900 |
| Rivaroxaban |  |  |  |  |  |  | 308 | 392 | 721 | 826 |
| Apixaban |  |  |  |  |  |  | 1022 | 504 | 707 | 420 |
| Argatroban |  | 6 |  | 18 |  | 20 |  | 16 |  | 38 |
| Heparin sodium |  | 5768 |  | 5799 |  | 5025 |  | 6010 |  | 7129 |
| LMWH |  | 33897 |  | 28824 |  | 31482 |  | 42435 |  | 56430 |
| Hospital H | | | | | | | | | | |
| Warfarin | 6045 | 1845 | 8845 | 1893 | 10819 | 2424 | 10170 | 1198 | 10537 | 1081 |
| Rivaroxaban |  |  |  |  | 283 | 379 | 3773 | 3358 | 6056 | 7739 |
| Apixaban |  |  |  |  | 169 | 76 | 1543 | 341 | 1297 | 373 |
| Edoxaban |  |  |  |  | 7 | 40 | 1455 | 172 | 3790 | 82 |
| Heparin sodium | 7610 | 22818 | 506 | 26070 | 426 | 32053 | 301 | 22796 | 201 | 28368 |
| LMWH | 18628 | 45165 | 953 | 55580 | 835 | 73879 | 640 | 74140 | 426 | 154772 |

Abbreviations: defined daily doses (DDDs); low molecular weight heparin (LMWH).
