# Supplementary material for: Anticoagulants utilization in eight hospitals within the Luzhou region from 2019 to 2023
Source: PLoS One. 2025 Jan 31;20(1):e0318463. doi: 10.1371/journal.pone.0318463 (PMC11785323; doi:10.1371/journal.pone.0318463)
Supplement: S3 Table — (DOCX) [file pone.0318463.s003.docx]

Supplementary Table 3: Inpatient and outpatient expenditure of different anticoagulants in eight hospitals.

|  | 2019 | | 2020 | | 2021 | | 2022 | | 2023 | |
| --- | --- | --- | --- | --- | --- | --- | --- | --- | --- | --- |
|  | Outpatient | Inpatient | Outpatient | Inpatient | Outpatient | Inpatient | Outpatient | Inpatient | Outpatient | Inpatient |
| Hospital A | | | | | | | | | | |
| Warfarin | 17.84 | 3.64 | 19.84 | 3.68 | 31.27 | 5.23 | 17.55 | 2.31 | 16.70 | 1.94 |
| Rivaroxaban | 264.75 | 251.05 | 268.78 | 258.73 | 302.67 | 279.77 | 129.17 | 101.54 | 124.50 | 105.05 |
| Apixaban |  |  |  |  | 0.17 |  |  |  |  |  |
| Edoxaban |  |  |  |  |  |  | 0.07 | 0.22 | 7.34 | 4.05 |
| Dabigatran ester | 0.17 | 0.14 | 6.11 | 1.43 | 6.35 | 0.39 | 18.24 | 3.27 | 16.17 | 1.66 |
| Heparin sodium | 0.04 | 12.08 | 0.10 | 15.41 | 0.12 | 14.09 | 0.14 | 10.05 | 0.13 | 8.83 |
| Heparin calcium |  |  |  |  |  |  |  | 39.80 | 0.17 | 429.23 |
| LMWH | 32.98 | 446.76 | 59.63 | 640.16 | 78.64 | 642.67 | 78.05 | 732.32 | 62.08 | 530.93 |
| Fondaparinux sodium |  |  | 0.42 | 6.27 | 11.35 | 41.65 | 37.08 | 7.50 | 16.75 | 0.35 |
| Bivarudin |  | 0.73 |  | 27.15 |  | 81.87 |  | 136.76 |  | 164.68 |
| Argatroban |  | 8.39 | 0.24 | 28.79 |  | 25.10 |  | 1.70 |  | 0.99 |
| Hospital B | | | | | | | | | | |
| Warfarin | 3.16 | 0.99 | 2.61 | 1.09 | 2.66 | 1.00 | 1.93 | 0.47 | 1.60 | 0.32 |
| Rivaroxaban | 54.88 | 20.51 | 76.49 | 42.11 | 80.53 | 41.98 | 14.75 | 7.14 | 9.46 | 4.62 |
| Apixaban |  |  | 0.38 | 1.84 | 0.41 | 2.01 | 0.31 | 2.92 | 5.04 | 8.08 |
| Edoxaban |  |  |  |  |  |  |  |  | 0.28 | 0.20 |
| Dabigatran ester | 8.96 | 2.47 | 3.93 | 5.11 | 29.10 | 8.93 | 10.47 | 1.41 | 8.39 | 0.58 |
| Heparin sodium | 0.30 | 18.91 | 0.18 | 8.66 | 0.08 | 4.58 | 0.01 | 6.84 | 0.04 | 10.18 |
| LMWH | 1.13 | 337.15 | 2.89 | 375.74 | 4.12 | 384.42 | 7.95 | 279.21 | 21.5 | 227.14 |
| Hospital C | | | | | | | | | | |
| Warfarin | 0.31 | 0.33 | 0.51 | 0.29 | 0.70 | 0.21 | 0.75 | 0.16 | 0.86 | 0.12 |
| Rivaroxaban |  | 0.08 | 0.79 | 1.16 | 9.28 | 9.41 | 6.79 | 10.28 | 7.08 | 7.27 |
| Bivarudin |  |  |  |  |  | 3.34 |  | 26.36 |  | 0.16 |
| Heparin sodium | 0.05 | 5.22 | 0.01 | 10.25 | 0.02 | 26.12 | 0.03 | 20.66 | 0.16 | 20.55 |
| Heparin calcium |  |  |  |  |  |  |  | 0.91 |  | 14.70 |
| LMWH | 0.12 | 56.63 | 0.43 | 61.77 | 0.81 | 84.57 | 4.41 | 127.3 | 3.312 | 97.9 |
| Hospital D | | | | | | | | | | |
| Warfarin | 1.70 | 0.74 | 2.78 | 1.17 | 2.89 | 1.07 | 1.78 | 0.40 | 1.54 | 0.32 |
| Rivaroxaban |  |  |  |  | 3.94 | 4.16 | 4.42 | 2.00 | 7.80 | 3.98 |
| Heparin sodium | 0.35 | 2.64 | 0.91 | 6.61 | 1.11 | 8.92 | 1.10 | 11.63 | 0.29 | 13.36 |
| LMWH |  | 134.77 | 0.30 | 256.83 |  | 283.33 | 0.61 | 266.42 |  | 118.62 |
| Hospital E | | | | | | | | | | |
| Warfarin | 1.05 | 0.29 | 1.47 | 0.27 | 1.54 | 0.30 | 1.29 | 0.24 | 1.12 | 0.22 |
| Rivaroxaban | 2.94 | 3.91 | 8.89 | 9.09 | 14.75 | 9.50 | 2.59 | 2.18 | 4.36 | 2.84 |
| Dabigatran ester | 0.13 |  |  |  | 0.13 |  | 0.34 |  | 0.31 |  |
| Heparin sodium |  | 7.95 | 0.01 | 7.95 | 0.01 | 7.81 | 0.05 | 7.18 | 0.04 | 7.67 |
| Heparin calcium | 0.01 | 11.22 | 0.01 | 8.40 |  | 14.29 |  | 17.26 |  | 14.57 |
| LMWH | 0.10 | 21.01 | 0.08 | 38.22 | 7.95 | 68.96 | 27.78 | 84.76 | 0.27 | 86.78 |
| Hospital F | | | | | | | | | | |
| Warfarin | 0.70 | 0.05 | 0.45 | 0.05 | 0.63 | 0.04 | 0.50 | 0.04 | 0.56 | 0.03 |
| Rivaroxaban |  |  |  |  |  |  | 0.38 | 0.02 | 2.69 | 1.39 |
| Argatroban |  |  |  |  |  |  |  | 0.04 |  | 0.16 |
| Heparin sodium | 0.11 | 0.42 | 0.13 | 0.18 | 0.16 | 1.86 | 0.24 | 2.52 | 0.28 | 4.16 |
| LMWH | 0.01 | 11.30 | 0.10 | 10.03 | 0.02 | 16.45 | 0.02 | 34.73 | 0.16 | 34.90 |
| Hospital G | | | | | | | | | | |
| Warfarin | 0.04 | 0.10 | 0.16 | 0.40 | 0.33 | 0.46 | 0.29 | 0.28 | 0.17 | 0.13 |
| Rivaroxaban |  |  |  |  |  |  | 0.42 | 0.53 | 0.98 | 1.23 |
| Apixaban |  |  |  |  |  |  | 1.44 | 0.71 | 0.99 | 0.59 |
| Argatroban |  | 1.60 |  | 4.77 |  | 5.36 |  | 0.56 |  | 0.76 |
| Heparin sodium |  | 3.55 |  | 4.55 |  | 3.94 |  | 4.71 |  | 5.59 |
| LMWH |  | 54.16 |  | 45.88 |  | 52.60 |  | 73.24 |  | 44.23 |
| Hospital H | | | | | | | | | | |
| Warfarin | 0.64 | 0.19 | 0.94 | 0.20 | 1.09 | 0.25 | 0.92 | 0.11 | 0.95 | 0.10 |
| Rivaroxaban |  |  |  |  | 0.39 | 0.52 | 5.15 | 4.58 | 8.27 | 10.56 |
| Apixaban |  |  |  |  | 0.24 | 0.11 | 2.18 | 0.48 | 0.95 | 0.08 |
| Edoxaban |  |  |  |  | 0.01 | 0.09 | 3.10 | 0.37 | 8.07 | 0.17 |
| Heparin sodium | 4.02 | 12.05 | 0.33 | 18.36 | 0.33 | 25.13 | 0.24 | 17.87 | 0.16 | 22.24 |
| LMWH | 14.65 | 35.54 | 0.75 | 43.68 | 0.66 | 67.55 | 0.63 | 98.31 | 0.32 | 93.87 |

Abbreviations: low molecular weight heparin (LMWH).
