# Supplementary material for: Anticoagulants utilization in eight hospitals within the Luzhou region from 2019 to 2023
Source: PLoS One. 2025 Jan 31;20(1):e0318463. doi: 10.1371/journal.pone.0318463 (PMC11785323; doi:10.1371/journal.pone.0318463)

PLOS ONE

Analysis of anticoagulant drug utilization in eight hospitals within the Luzhou region from 2019 to 2023

--Manuscript Draft--

**Manuscript Number:**	PONE-D-24-36242
**Article Type:**	Research Article
**Full Title:**	Analysis of anticoagulant drug utilization in eight hospitals within the Luzhou region from 2019 to 2023
**Short Title:**	Analysis of anticoagulant drug
**Corresponding Author:**	Hongli Luo<br>The Affiliated Hospital of Southwest Medical University<br>Luzhou, Sichuan CHINA
**Keywords:**	anticoagulant drug, drug utilization, defined daily doses, expenditure
**Abstract:**	Background: The objective of this retrospective study was to assess the usage patterns of anticoagulant drugs within a specific region over recent years, analyze any discernible trends, and provide valuable insights for their management and rational use.<br>Methods: The usage of anticoagulant drugs was retrospectively analyzed using descriptive statistical methods. Data on the utilization of anticoagulant drugs in tertiary hospital within a district from 2019 to 2023 were collected, and the expenditure, defined daily doses (DDDs), and defined daily dose cost (DDDC) were calculated. Diagrams illustrating the trend of change were generated.<br>Results: From 2021 to 2023, the DDDs of rivaroxaban and low molecular weight heparin (LMWH) exhibited an annual increase across the eight hospitals. However, the expenditure of rivaroxaban and LMWH showed a downward trend in 2021 and 2022. The DDDC of rivaroxaban decreased significantly from 55.20 CNY in 2019 to 4.28 CNY in 2023, while for LMWH it decreased from 19.68 CNY in 2019 to 12.67 CNY in 2023. In contrast, the DDDs of warfarin and heparin sodium remained relatively stable over the five-year period. Furthermore, oral anticoagulants were predominantly prescribed for outpatient care, whereas LMWH was primarily chosen for inpatient treatment.<br>Conclusion: With the increasing number of VTE patients, there has been a corresponding rise in the utilization and expenditure on anticoagulant medications. Among various anticoagulants, rivaroxaban and LMWH have emerged as prominent choices due to their minimal adverse reactions and superior therapeutic efficacy.
**Order of Authors:**	Wei Luo
	Yan Li
	Jiali Yang
	Yang Liu
	Yue Shi
	Hongli Luo
**Additional Information:**	
**Question**	**Response**
**Financial Disclosure**<br><br>Enter a financial disclosure statement that describes the sources of funding for the work included in this submission. Review the submission guidelines for detailed requirements. View published research	Yes

Powered by Editorial Manager® and ProduXion Manager® from Aries Systems Corporation

articles from *PLOS ONE* for specific examples.<br><br>This statement is required for submission and **will appear in the published article** if the submission is accepted. Please make sure it is accurate.<br><br>**Funded studies**<br>Enter a statement with the following details:<br>• Initials of the authors who received each award<br>• Grant numbers awarded to each author<br>• The full name of each funder<br>• URL of each funder website<br>• Did the sponsors or funders play any role in the study design, data collection and analysis, decision to publish, or preparation of the manuscript?<br><br>Did you receive funding for this work?	
Please add funding details.<br>as follow-up to "**Financial Disclosure**<br><br>Enter a financial disclosure statement that describes the sources of funding for the work included in this submission. Review the submission guidelines for detailed requirements. View published research articles from *PLOS ONE* for specific examples.<br><br>This statement is required for submission and **will appear in the published article** if the submission is accepted. Please make sure it is accurate.	Sichuan Provincial Department of Science and Technology project (No. 2023JDKP0040)

**Funded studies**<br>Enter a statement with the following details:<br>• Initials of the authors who received each award<br>• Grant numbers awarded to each author<br>• The full name of each funder<br>• URL of each funder website<br>• Did the sponsors or funders play any role in the study design, data collection and analysis, decision to publish, or preparation of the manuscript?<br><br>Did you receive funding for this work?"	
Please select the country of your main research funder (please select carefully as in some cases this is used in fee calculation).<br>as follow-up to "**Financial Disclosure**<br><br>Enter a financial disclosure statement that describes the sources of funding for the work included in this submission. Review the submission guidelines for detailed requirements. View published research articles from *PLOS ONE* for specific examples.<br><br>This statement is required for submission and **will appear in the published article** if the submission is accepted. Please make sure it is accurate.<br><br>**Funded studies**<br>Enter a statement with the following details:<br>• Initials of the authors who received each award<br>• Grant numbers awarded to each author<br>• The full name of each funder<br>• URL of each funder website<br>• Did the sponsors or funders play any role in the study design, data collection and analysis, decision to publish, or preparation of the manuscript?<br><br>Did you receive funding for this work?"	CHINA - CN
**Competing Interests**	The authors declare there are no conflicts of interest.

Use the instructions below to enter a competing interest statement for this submission. On behalf of all authors, disclose any competing interests that could be perceived to bias this work—acknowledging all financial support and any other relevant financial or non-financial competing interests.<br><br>This statement is required for submission and **will appear in the published article** if the submission is accepted. Please make sure it is accurate and that any funding sources listed in your Funding Information later in the submission form are also declared in your Financial Disclosure statement.<br><br>View published research articles from *PLOS ONE* for specific examples.<br><br>**NO authors have competing interests**<br><br>Enter: *The authors have declared that no competing interests exist.*<br><br>**Authors with competing interests**<br><br>Enter competing interest details beginning with this statement:<br><br>*I have read the journal's policy and the authors of this manuscript have the following competing interests: [insert competing interests here]*<br><br>* typeset	
**Ethics Statement**<br><br>Enter an ethics statement for this submission. This statement is required if the study involved:<br><br>• Human participants<br>• Human specimens or tissue<br>• Vertebrate animals or cephalopods<br>• Vertebrate embryos or tissues	The data used in this study were completely anonymized, did not require review and approval by ethics committees, and the requirement for informed consent was waived.

Powered by Editorial Manager® and ProduXion Manager® from Aries Systems Corporation

• Field research<br><br>Write "N/A" if the submission does not require an ethics statement.<br><br>General guidance is provided below. Consult the submission guidelines for detailed instructions. **Make sure that all information entered here is included in the Methods section of the manuscript.**	

Powered by Editorial Manager® and ProduXion Manager® from Aries Systems Corporation

**Format for specific study types**<br><br>**Human Subject Research (involving human participants and/or tissue)**<br>• Give the name of the institutional review board or ethics committee that approved the study<br>• Include the approval number and/or a statement indicating approval of this research<br>• Indicate the form of consent obtained (written/oral) or the reason that consent was not obtained (e.g. the data were analyzed anonymously)<br><br>**Animal Research (involving vertebrate animals, embryos or tissues)**<br>• Provide the name of the Institutional Animal Care and Use Committee (IACUC) or other relevant ethics board that reviewed the study protocol, and indicate whether they approved this research or granted a formal waiver of ethical approval<br>• Include an approval number if one was obtained<br>• If the study involved *non-human primates*, add *additional details* about animal welfare and steps taken to ameliorate suffering<br>• If anesthesia, euthanasia, or any kind of animal sacrifice is part of the study, include briefly which substances and/or methods were applied<br><br>**Field Research**<br><br>Include the following details if this study involves the collection of plant, animal, or other materials from a natural setting:<br>• Field permit number<br>• Name of the institution or relevant body that granted permission	
**Data Availability**<br><br>Authors are required to make all data underlying the findings described fully available, without restriction, and from the time of publication. PLOS allows rare exceptions to address legal and ethical concerns. See the PLOS Data Policy and FAQ for detailed information.	No - some restrictions will apply

A Data Availability Statement describing where the data can be found is required at submission. Your answers to this question constitute the Data Availability Statement and **will be published in the article**, if accepted.<br><br>**Important:** Stating 'data available on request from the author' is not sufficient. If your data are only available upon request, select 'No' for the first question and explain your exceptional situation in the text box.<br><br>Do the authors confirm that all data underlying the findings described in their manuscript are fully available without restriction?	
**Describe where the data may be found in full sentences. If you are copying our sample text, replace any instances of XXX with the appropriate details.**<br><br>• If the data are **held or will be held in a public repository**, include URLs, accession numbers or DOIs. If this information will only be available after acceptance, indicate this by ticking the box below. For example: *All XXX files are available from the XXX database (accession number(s) XXX, XXX.).*<br>• If the data are all contained **within the manuscript and/or Supporting Information files**, enter the following: *All relevant data are within the manuscript and its Supporting Information files.*<br>• If neither of these applies but you are able to provide **details of access elsewhere**, with or without limitations, please do so. For example:<br><br>*Data cannot be shared publicly because of [XXX]. Data are available from the XXX Institutional Data Access / Ethics Committee (contact via XXX) for researchers who meet the criteria for access to confidential data.*<br><br>*The data underlying the results presented in the study are available from (include the name of the third party*	The data that support the findings of this study are available from Hospital Information System and Prescription Automatic Screening System of Sichuan Medico Software Research and Development Co., Ltd. but restrictions apply to the availability of these data, which were used under license for the current study, and so are not publicly available. Data are however available from the authors upon reasonable request and with permission of Hospital Information System and Prescription Automatic Screening System of Sichuan Medico Software Research and Development Co., Ltd.

and contact information or URL).<br>• This text is appropriate if the data are owned by a third party and authors do not have permission to share the data.<br><br>* typeset	
Additional data availability information:	

Manuscript Click here to access/download;Manuscript;manuscript.docx

Analysis of anticoagulant drug utilization in eight hospitals within the Luzhou region from 2019 to 2023

Wei Luo[1,2#], Yan Li[3#], Jiali Yang[1,2#], Yang Liu[1,2], Yue Shi[1,2], Hongli Luo[1*]
1. Department of Pharmacy, The Affiliated Hospital of Southwest Medical University, Luzhou, China
2. School of Pharmacy, Southwest Medical University, Luzhou, China
3. Department of Pharmacy, The First Afflicted Hospital of Chengdu Medical College, Chengdu, China
#Wei Luo, Yan Li and Jiali Yang contributed equally to this work.
* lyfylhl@163.com

Abstract

Background: The objective of this retrospective study was to assess the usage patterns of anticoagulant drugs within a specific region over recent years, analyze any discernible trends, and provide valuable insights for their management and rational use.
Methods: The usage of anticoagulant drugs was retrospectively analyzed using descriptive statistical methods. Data on the utilization of anticoagulant drugs in tertiary hospital within a district from 2019 to 2023 were collected, and the expenditure, defined daily doses (DDDs), and defined daily dose cost (DDDC) were calculated. Diagrams illustrating the trend of change were generated.
Results: From 2021 to 2023, the DDDs of rivaroxaban and low molecular weight heparin (LMWH) exhibited an annual increase across the eight hospitals. However, the expenditure of rivaroxaban and LMWH showed a downward trend in 2021 and 2022. The DDDC of rivaroxaban decreased significantly from 55.20 CNY in 2019 to 4.28 CNY in 2023, while for LMWH it decreased from 19.68 CNY in 2019 to 12.67 CNY in 2023. In contrast, the DDDs of warfarin and heparin sodium remained relatively stable over the five-year period. Furthermore, oral anticoagulants were predominantly prescribed for outpatient care, whereas LMWH was primarily chosen for inpatient treatment.
Conclusion: With the increasing number of VTE patients, there has been a corresponding rise in the utilization and expenditure on anticoagulant medications. Among various anticoagulants, rivaroxaban and LMWH have emerged as prominent choices due to their minimal adverse reactions and superior therapeutic efficacy.
Keywords: anticoagulant drug, drug utilization, defined daily doses, expenditure

Introduce

Venous thromboembolism (VTE) is a prevalent cardiovascular disorder characterized by the formation of blood clots in the lower extremity veins, which subsequently dislodge and migrate to the heart and lungs. This condition encompasses two main entities: deep vein thrombosis (DVT) and pulmonary embolism (PE)[1]. The incidence of VTE affects millions of individuals worldwide annually, leading to significant morbidity and mortality as same as myocardial infarction[2]. In Europe and the United States, the estimated VTE incidence is approximately 1-2 per 1000 individuals. Comparatively, Asia exhibits a lower rate of VTE compared to Europe and the United States[3]. Notably in China, there has been a consistent increase in prioritizing VTE prevention as one of the top ten national medical safety improvement goals for four consecutive years. The prevention of VTE is feasible. However, the presence of obesity, diabetes, smoking, hypertension and hyperlipidemia can significantly elevate the risk of VTE[4,5]. Approximately 30% of patients diagnosed with VTE experience recurrence within a decade[6]. Furthermore, VTE not only poses a risk of mortality in the first year following diagnosis but also remains a significant cause of death during the subsequent 30-year follow-up period. Mortality from VTE and other cardiovascular, cancer, and respiratory diseases is significantly higher in patients with VTE compared with the general population[7].

Anticoagulant therapy constitutes the cornerstone in the management of VTE. VTE in the course of the disease is usually divided into three different stages: an initial acute phase lasting for 5-10 days following the diagnosis of VTE, a subsequent maintenance phase spanning 3-6 months, and finally an extended phase extending beyond this designated timeframe. Diverse stages entail distinct demands for the utilization of anticoagulant medications[4,8]. Anticoagulant medications can be broadly classified into three categories: vitamin K antagonists (VKA), direct oral anticoagulants (DOACs), and parenteral anticoagulants. Clinical indications for each anticoagulant should be considered based on the risk of adverse drug events, a clear index, drug treatment mechanism (hepatic and renal metabolism), variable drug half-life, treatment requirements, monitoring needs[9,10].

The use of anticoagulant drugs when the main adverse reaction is increased risk of bleeding[11]. In a meta-analysis of 33 studies involving 4374 patients receiving oral anticoagulant therapy, the case fatality rate for major bleeding was determined to be 13.4% (95% CI, 9.4% to 17.4%)[12]. VKA drugs, exemplified by warfarin, exhibit a narrow therapeutic index necessitating regular monitoring of patient prothrombin time to maintain an international normalized ratio (INR) within the range of 2.0 to 3.0[13]. Over a span of five years, a prospective study on warfarin demonstrated that by adjusting the treatment strategy based on INR, the incidence rate of adverse drug reactions (ADRs) associated with warfarin significantly decreased from 3.8% to 0.98% ($p < 0.0001$)[14]. The primary guideline recommendation is to prioritize DOACs for the treatment of VTE, as opposed to the vitamin K antagonist, due to DOACs exemption from regular INR monitoring[15]. Furthermore, DOACs exhibit a low risk of bleeding and drug interactions, while also demonstrating minimal impact on food-drug interactions[4,16].

With the publication of “Good Manufacturing Practice”, medical institutions have increasingly prioritized drug quality control, encompassing not only pharmaceutical production, drug procurement, and storage but also rational clinical drug utilization. By fortifying quality supervision,

the standard of rational drug use is enhanced. The field of drug utilization research (DUR) employs a range of descriptive and analytical methods to quantitatively assess, comprehend, and evaluate the processes involved in drug prescribing, dispensing, and consumption. Furthermore, DUR serves as a platform for testing intervention measures aimed at enhancing the quality of these processes[17]. In 1981, the World Health Organization (WHO) adopted Defined Daily Dose (DDD) as a standardized unit of measurement for drugs. DUR is employed to identify early indicators of inappropriate drug utilization in order to enhance the safety and efficacy of drug usage within populations, thereby augmenting the overall quality of pharmaceutical administration[18].

This study aims to luzhou area through the analysis of the drug utilization research in tertiary hospital nearly five years of anticoagulant drugs usage, combining economics discussion and thinking of anticoagulant drugs selection and reasonable use.

Method

Data collection

This study was a retrospective multicenter study in Luzhou City, and data collection began in March 2024. We extracted data related to anticoagulant drugs from the Hospital Information System (HIS) of eight hospitals in Luzhou, China, categorized as Third-Level Grade A (A, B) and Third-Level Grade B (C, D, E, F, G, H), for the period January 2019 to December 2023. The extracted data includes prescription drugs' general and commodity names, medication specifications, quantities, and amounts. The analysis encompassed VKA (Warfarin), DOACs (rivaroxaban, apixaban, dabigatran etexilate, etc.), and parenteral anticoagulants (heparins, fondaparinux, argatroban, etc.). Information on individual participants could not be accessed and identified during and after data collection.

Ethics statement

The data used in this study were completely anonymized, did not require review and approval by ethics committees, and the requirement for informed consent was waived.

Study design

The present study employed descriptive statistics to retrospectively examine the utilization of anticoagulant medications across various hospitals in Luzhou from January 2019 to December 2023. After data collection, types of drugs, expenditure, defined daily doses (DDDs), defined daily dose cost (DDDC), such as statistics, and compare the use present situation and trend of anticoagulant drugs. Among them, heparins with lower molecular weight such as enoxaparin, nadroparin and dalteparin are uniformly referred to as low molecular weight heparin (LMWH).

The expenditure was determined using the Chinese currency Renminbi "yuan" (CNY) to determine the expenditure of anticoagulant drugs, and 7.27 CNY was equal to 1 US dollar. The DDD method was used to calculate the DDDs. Referring to the DDD values provided by WHO, the DDD values that could not be searched were implemented according to the management scheme suggested in the manufacturer's instructions approved by the Food and Drug Administration. DDDs= annual consumption of a drug/DDD value of the drug. The DDDs are primarily employed for drug utilization assessment. A higher value of DDDs indicates a greater frequency of drug use, thereby reflecting a clinical inclination towards the choice of the drug. DDDC = an annual consumption amount of the drug/drug DDDs value. The DDDC index was previously utilized to reflect the mean daily expenditures of patients on specific medications, with a higher DDDC value indicating a greater economic burden on patients.

The selection of warfarin as a prescription was made to assess the rationality of its usage. The warfarin leaflet and “China expert consensus of warfarin anticoagulant therapy”, "Guidelines on oral anticoagulation with warfarin – fourth edition" serve as the standard for evaluating indications of warfarin use.

The statistical indicators of eight hospitals in Luzhou were calculated using Excel software. For substances with different specifications and manufacturers, the quantities were standardized through unit conversion or summation to obtain the total amount of the drug. Subsequently, Graphpad Prism 8.0 software was used to produce graphs.

Results

Different hospital drug use

The current landscape of anticoagulant drugs encompasses various categories, including parenteral anticoagulants, VKA, and DOACs. In recent years, the utilization of anticoagulant drugs in luzhou eight hospitals reveals (Figure 1) that the predominant anticoagulants employed are warfarin, rivaroxaban, heparin sodium, and LMWH. While other anticoagulant drugs are being used, it can be observed that Third-Level Grade A hospitals prescribing various medications exceeds that of Third-Level Grade B hospitals. Considering the different types of drugs used in different hospitals, four anticoagulant drugs, warfarin, rivaroxaban, heparin sodium and low molecular weight heparin, which were used in eight hospitals were selected as the subjects of follow-up study.

Figure 1: usage of anticoagulant drugs at eight hospitals. DDDs: defined daily doses; LMWH: low molecular weight heparin.

Overall trends in anticoagulants

The prevalence of VTE patients has been on the rise, consequently elevating the significance of anticoagulant medications. The DDDs of rivaroxaban and LMWH exhibited a consistent upward trend from 2019 to 2023 across eight hospitals (Figure 2b, 2d). The utilization of rivaroxaban in Third-Level Grade B hospitals was minimal during the period from 2019 to 2020, with significant adoption observed only between 2021 and 2023. H hospital, for example, in 2019 and 2020 did not utilize rivaroxaban, however, starting from 2021, the utilization of DDDs increased significantly from 662 to 13,795 in the subsequent years. The DDDs of warfarin have exhibited relatively stable trends in the past five years across eight hospitals. However, heparin sodium showed different trends in the eight hospitals. Hospital A exhibited a declining trend over the years, while hospitals B, E, G, and H demonstrated relatively stable patterns. Conversely, hospitals C, D, and F displayed an upward trend year after year. Furthermore, both hospitals C and H administered higher dosages of heparin sodium compared to the first-class hospitals A and B.

Figure 2: Utilization of anticoagulants from 2019 to 2023. a: warfarin. b: rivaroxaban; c: heparin sodium; d: LMWH. DDDs: defined daily doses; LMWH: low molecular weight heparin.

The comparison of anticoagulant drug usage and expenditure amounts reveals a similar trend in the sum of warfarin and heparin sodium expenditure, as well as changes in DDDs (Figure 3a, 3c). Notably, the expenditure of rivaroxaban and LMWH exhibited divergent trends in terms of DDDs. For rivaroxaban, the utilization of DDDs increased across the eight hospitals from 2019 to 2023; however, there was a decrease in expenditure observed from 2021 to 2023 (Figure 3b). Similarly, the expenditure of LMWH exhibit a decreasing trend in 2022-2023 (Figure 3d). In A hospital, the DDDs for rivaroxaban increased from 128,232 in 2021 to 358,435 in 2023. Additionally, the corresponding expenditure decreased from 5.8244 million CNY in 2021 to 2.2955 million CNY in

2023.

Figure 3: The expenditure on anticoagulant drugs in the period of 2019-2023(million CNY). a: Warfarin; b: Rivaroxaban; c: Heparin sodium; d: LMWH. LMWH: low molecular weight heparin.

The difference of anticoagulant drugs outpatient and inpatient

In the past five years, there has been a noticeable upward trend in the number of outpatient visits and inpatient. In all eight hospitals, warfarin, rivaroxaban, and LMWH were the predominant anticoagulant drugs administered in the outpatient department, with their utilization significantly surpassing that of heparin sodium (Figure 4). Despite the high dosage of LMWH in terms of DDDs, there were still discernible distinctions compared to the overall quantity. Taking Hospital A as an example, the outpatient DDDs of warfarin, rivaroxaban, and LMWH in 2023 were recorded as 185,580, 237,453, and 46,401 respectively, which accounting for 89.6%, 66.2% and 13.4% of the total DDDs in the hospital. This finding also indicates the prevailing utilization of oral anticoagulants as the primary choice for outpatient anticoagulation therapy.

Figure 4: Outpatient utilization of anticoagulants from 2019 to 2023. a: Warfarin; b: Rivaroxaban; c: Heparin sodium; d: LMWH. DDDs: defined daily doses; LMWH: low molecular weight heparin.

The administration of LMWH in inpatient surpasses that of the other three drugs by a significant margin (Figure 5). Moreover, a substantial escalation in the utilization of heparin sodium was observed within the hospital setting when compared to DDDs of outpatient heparin sodium. Taking Hospital A as an example, the hospitalization DDDs of heparin sodium and LMWH in 2023 were 11,269 and 300,236, respectively, accounting for 98.6% and 86.6% of the total DDDs administered at the hospital. Furthermore, the findings indicate a prevailing utilization of parenteral anticoagulants in hospital settings for the administration of anticoagulant drugs. Rivaroxaban also holds a significant position among inpatient anticoagulant medications in hospital A, accounting for 33.8% of the total in 2023.

Figure 5: Inpatient utilization of anticoagulants from 2019 to 2023. a: Warfarin; b: rivaroxaban; c: Heparin sodium; d: LMWH. DDDs: defined daily doses; LMWH: low molecular weight heparin.

Due to the substantial volume of prescriptions in both outpatient and inpatient settings, the rational utilization of medications has emerged as a significant concern. The prescription of warfarin in hospital A was used as an exemplification to comprehend the indications for warfarin administration. Warfarin is the most widely utilized oral anticoagulant globally for the prevention and treatment of various thrombotic events, including deep-vein thrombosis, atrial fibrillation, valvular heart disease and prosthetic valves, as well as peripheral vascular disease[19]. In 2023, the retrieval of warfarin prescriptions was categorized into inpatient (n=734) and outpatient (n=6224) prescriptions. According to the prescription diagnosis, the classification includes mechanical valve replacement status, atrial fibrillation, venous thrombosis, cerebral infarction, cardiac surgery (valvular surgery, implantable cardioverter defibrillator (ICD), Bentall), heart valve disease and other indications (Table 1). The utilization of warfarin in the outpatient setting predominantly pertained to mechanical valve replacement (74.1%), followed by atrial fibrillation (20.6%). However, the utilization of warfarin in the inpatient setting predominantly pertained to cardiac valvular disease (54.4%), followed by atrial fibrillation (46.0%). Due to a prescription may contain a variety of indications, so these prescriptions will be superposition calculation.

Table 1: Classification of indications for warfarin prescription

	Outpatient	Inpatient
Mechanical valve replacement	4612	92

Atrial fibrillation	1285	338
Venous thrombus	53	36
Cerebral infarction	483	161
Postoperative state	816	77
Valvular heart disease	428	399
Others	75	119

DDDC of anticoagulant drugs

The DDDC represents the average daily cost value, and a higher DDDC indicates increased economic burden on patients. DDDC value is the lowest drug warfarin, nearly five years are about 1 CNY, belong to the burden lighter (figure 6). And the DDDC value of rivaroxaban, heparin sodium, and LMWH is relatively high. Among others, the DDDC value of rivaroxaban exhibited a decline starting from 2021, with a decrease from 55.20 CNY in 2019 to 4.28 CNY in 2023. Similarly, the DDDC value of LMWH began to decline in 2023, from 19.68 CNY in 2019 to 12.67 CNY in 2023. However, The DDDC values of warfarin and heparin sodium have exhibited a relatively stable trend over the past five years.

Figure 6: DDDC for four Anticoagulants from 2019 to 2023. DDDC: defined daily dose cost.

Discussion

VTE represents a significant healthcare concern with potentially grave implications, and the occurrence of acute VTE can lead to fatal outcomes. Hospitalized surgical or medical patients are particularly susceptible to VTE, and this issue persists even after discharge from the hospital[20–22]. An estimated 370,000 deaths related to VTE occur annually in six European countries, with three-quarters attributed to hospital-acquired VTE[23]. VTE can be divided into two parts: prevention and treatment. Patients eligible for VTE prophylaxis may receive anticoagulant medications (such as rivaroxaban, warfarin, or heparin) to effectively impede blood coagulation, provided that contraindications have been excluded. In addition, can also be in patients with VTE by grade of wear compression stockings, early to walk and exercise to prevent VTE, such as foot and ankle[24]. Rivaroxaban can be employed as a standalone therapeutic agent for the management of VTE. Warfarin and dabigatran etexilate may need to be done after use for a period of time of parenteral anticoagulants[25].

The primary focus of outpatient clinics lies in the management of mild or chronic diseases, wherein patients are typically prescribed self-administered medications. Oral administration is safe and convenient. Hence, oral anticoagulant medications such as warfarin and rivaroxaban constitute the primary pharmacological agents for anticoagulation therapy in outpatient settings across eight hospitals. Furthermore, this study revealed a substantial elevation in the outpatient clinic regarding the dosage of LMWH. In a nationwide survey and a randomized, controlled trial, outpatient prophylaxis with LMWH demonstrated efficacy in mitigating the risk of VTE among patients with immobilization of the lower extremities[26,27]. The primary purpose of hospitalization is to address moderate to severe diseases that necessitate systemic treatment, and the treatment period is long. In hospitalized patients, intravenous administration is frequently necessary to promptly alleviate patient symptoms. Furthermore, considering the impaired gastrointestinal drug absorption in certain patients due to underlying diseases, parenteral administration becomes the sole viable option. Consequently, LMWH surpasses other anticoagulants in terms of its utilization as an anticoagulant therapy for hospitalized patients. The selection of different drugs in various environments can

effectively demonstrate the role of drug dosage forms and enhance drug efficacy.

The market has become highly competitive with the introduction of DOACs, and warfarin is on the brink of elimination due to its adverse effects and poor compliance[28]. According to the 2021 "Antithrombotic treatment of VTE disease: Second Update of the Chest Guideline and Expert Panel report"[29], DOACs including apixaban, dabigatran, edoxaban, or rivaroxaban are recommended as superior alternatives to warfarin for initial anticoagulation in patients with VTE. For patients who are contraindicated for DOACs, long-term administration of warfarin is recommended as an alternative anticoagulation strategy. Therefore, based on the DDDs of warfarin and rivaroxaban over the past 5 years (Figure 2), it can be observed that the DDDs of warfarin have exhibited a consistent trend, with certain hospitals even experiencing a decline. However, there has been a significant upward trend in the DDDs of rivaroxaban across the eight hospitals since 2021. Furthermore, warfarin exhibits a lightly worse compared to DOACs in terms of adverse effects and clinical efficacy. Patients receiving DOACs exhibited a significantly reduced risk of disease progression and a lower frequency of bleeding events compared to those administered warfarin[30–33]. The adherence to warfarin is suboptimal due to its adverse effects and the necessity for frequent monitoring of INR values. The study demonstrated a significantly higher treatment adherence rate among rivaroxaban users compared to apixaban users (AD: 5.8%; $P<0.001$), dabigatran users (AD: 9.5%; $P<0.001$), and warfarin users (AD: 13.6%; $P<0.001$[34]. Rivaroxaban does not necessitate routine monitoring, exhibits a lower incidence of adverse drug reactions, and is unencumbered by vitamin K-rich foods, rendering it the preferred anticoagulant over warfarin[35].

Although DOACs have demonstrated noninferiority to warfarin in patients with atrial fibrillation[36,37], the standard of care for patients with rheumatic mitral stenosis and those undergoing mechanical valve replacement still remains lifelong warfarin use[38]. Furthermore, post cardiac valve surgery, warfarin is typically prescribed for patients[39]. Regarding the diagnoses included in this study, the overwhelming majority of them fulfill the indication criteria for warfarin therapy. According to the expert consensus statement on ICD therapy[40], administration of warfarin for anticoagulation purposes is not recommended following ICD surgery. Therefore, the prescription of warfarin following ICD implantation is deemed irrational; however, it constitutes a negligible proportion. Despite its flaws, warfarin is generally prescribed reasonably. However, collaborative efforts are still required to further enhance the rationality of prescribing practices.

Heparin is one of the commonly used anticoagulants for a significant number of individuals. With the extensive investigation of their pharmacological effects, heparins have also been employed in the treatment of various other diseases. The anticoagulant effect of LMWH is accompanied by its additional properties including anti-inflammatory, antiviral, angiogenesis inhibition, and cell adhesion inhibition[41]. Even the novel coronavirus that emerged in the past few years has a certain effect. Studies have demonstrated that novel coronavirus is capable of inducing pulmonary injury, microvascular endothelial damage, and ultimately thrombosis[42]. The transmission of novel coronavirus is anticipated to result in an upsurge in the demand for heparin medications. Moreover, intravenous access is frequently established in hospitalized patients, and heparin serves as our standard of care for the prevention of venous access thrombosis and the mitigation of catheter-related infections[43].

The classification of heparin includes unfractionated heparin and LMWH. There are two formulations available for unfractionated heparin, namely heparin sodium and heparin calcium. The utilization of heparin sodium was significantly higher than that of heparin calcium in our study

conducted across eight hospitals (Figure 1). According to a study conducted by Lee-White Clotting Time (LWCT), activated partial thromboplastin time (APTT), and thrombin calcium clotting time (TCCT) measurements, subcutaneous injection of both heparin sodium and heparin calcium induced similar anticoagulant responses in normal subjects[44]. However, DDDC of heparin sodium (9.54 CNY) was significantly lower than that of heparin calcium (29.12 CNY). In order to alleviate the financial burden on patients, heparin sodium is prescribed more frequently. LMWH because of its less side effects and longer half-life, making it more widely used in hospital and external environment[45,46]. The pooled analysis involving 1398 postoperative participants demonstrated a significant reduction in the risk of heparin-induced thrombocytopenia with LMWH compared to unfractionated heparin (RR: 0.23, 95%CI: 0.07 to 0.73)[45]. The DDDs of heparin sodium exhibited an uneven trend across eight hospitals over the past 5 years, whereas LMWH demonstrated a consistent year-on-year increase. These findings suggest that unfractionated heparin is being gradually replaced by LMWH.

This study revealed an upward trend in the DDDs of rivaroxaban and LMWH over the past five years, whereas their consumption amount and DDDC exhibited a decline in 2021 and 2023, respectively. In order to ensure equitable accessibility to medications, China has initiated a novel nationwide centralized drug procurement program[47]. The inclusion of drugs in the centralized procurement framework leads to a significant reduction in their prices, thereby alleviating the financial burden on patients. This is the reason why drugs like rivaroxaban and LMWH have witnessed an increase in utilization while experiencing a decline in cost over recent years. Furthermore, a retrospective study demonstrated that the implementation of a centralized procurement policy resulted in significant enhancements in adherence to oral anticoagulation therapy and an upsurge in the proportion of patients opting for DOAC[48]. Despite the increasing utilization of DOACs in recent years, warfarin remains a pivotal therapeutic option for patients due to its cost-effectiveness.

The present study also possesses certain limitations. Firstly, the dataset used in this study exclusively comprises tertiary hospitals located in Luzhou. Unfortunately, statistics regarding the usage of anticoagulant drugs are not available for secondary hospitals and other hospital levels in Luzhou. However, we contend that tertiary hospitals can effectively serve as representative indicators of the medical and drug utilization landscape in Luzhou, thereby allowing for generalizability to other healthcare facilities across China. Secondly, the present study did not encompass the inclusion of antiplatelet medications and thrombolysis, which could potentially impact the analysis pertaining to the utilization of anticoagulant drugs. Thirdly, the retrospective nature of our study, as opposed to a randomized controlled trial, may limit the strength of our findings. Furthermore, our assessment solely focused on the appropriateness of warfarin prescription for its specific indication, without considering the suitability of prescribing alternative anticoagulants or the practices followed by other medical institutions in terms of anticoagulant prescriptions. Despite certain limitations, this study offers valuable insights into the contemporary utilization of anticoagulants. In future research endeavors, collaborative efforts between society and medical professionals are imperative to establish a comprehensive framework for the judicious utilization of this particular class of medications.

Conclusion

This study shows that between 2019 and 2023, the use and expenditure of anticoagulants

increased with the increase in VTE patients. Among them, the utilization of rivaroxaban and LMWH has exhibited a consistent upward trend in recent years owing to their minimal adverse reactions and remarkable therapeutic efficacy. The implementation of the centralized procurement policy has significantly reduced the prices of anticoagulant drugs, such as rivaroxaban and LMWH, thereby alleviating the economic burden on patients. This study examined the evolving trends in anticoagulant drug utilization, emphasizing the imperative of establishing a comprehensive VTE prevention and treatment network centered around primary healthcare institutions. Such an approach will effectively guide patients towards appropriate medical interventions while ensuring cost containment.

Acknowledgments

The authors thank the clinical pharmacists who collected the prescribing data in this study.

Data availability statement

The data that support the findings of this study are available from Hospital Information System and Prescription Automatic Screening System of Sichuan Medico Software Research and Development Co., Ltd. but restrictions apply to the availability of these data, which were used under license for the current study, and so are not publicly available. Data are however available from the authors upon reasonable request and with permission of Hospital Information System and Prescription Automatic Screening System of Sichuan Medico Software Research and Development Co., Ltd.

Funding information

This study was supported by Sichuan Provincial Department of Science and Technology project (No. 2023JDKP0040).

Competing interests:

The authors declare there are no conflicts of interest.

Author contributions

Conceptualization: Hongli Luo

Data curation: Wei Luo, Yan Li, Jiali Yang

Investigation: Yang Liu, Yue Shi

Methodology: Wei Luo, Hongli Luo

Visualization: Wei Luo, Yan Li, Jiali Yang

Writing– original draft: Wei Luo, Yan Li, Jiali Yang

Writing– review & editing: Wei Luo, Hongli Luo

Reference

1. Voukalis C, Lip GYH, Shantsila E. Non-vitamin K oral anticoagulants versus vitamin K antagonists in the treatment of venous thromboembolic disease. Expert Opin Pharmacother. 2016;17: 2033–2047. doi:10.1080/14656566.2016.1232393
2. Arnold MJ. Thromboembolic Disease. Prim Care: Clin Off Pract. 2024;51: 65–82. doi:10.1016/j.pop.2023.07.004
3. Lutsey PL, Zakai NA. Epidemiology and prevention of venous thromboembolism. Nat Rev Cardiol. 2023;20: 248–262. doi:10.1038/s41569-022-00787-6
4. Di Nisio M, Van Es N, Büller HR. Deep vein thrombosis and pulmonary embolism. Lancet. 2016;388: 3060–3073. doi:10.1016/S0140-6736(16)30514-1
5. Goldhaber SZ. Risk factors for venous thromboembolism. J Am Coll Cardiol. 2010;56: 1–7. doi:10.1016/j.jacc.2010.01.057
6. Heit JA, Spencer FA, White RH. The epidemiology of venous thromboembolism. J Thromb Thrombolysis. 2016;41: 3–14. doi:10.1007/s11239-015-1311-6
7. Søgaard KK, Schmidt M, Pedersen L, Horváth–Puhó E, Sørensen HT. 30-year mortality after venous thromboembolism: a population-based cohort study. Circulation. 2014;130: 829–836. doi:10.1161/CIRCULATIONAHA.114.009107
8. Streiff MB, Agnelli G, Connors JM, Crowther M, Eichinger S, Lopes R, et al. Guidance for the treatment of deep vein thrombosis and pulmonary embolism. J Thromb Thrombolysis. 2016;41: 32–67. doi:10.1007/s11239-015-1317-0
9. McRae HL, Militello L, Refaai MA. Updates in Anticoagulation Therapy Monitoring. Biomedicines. 2021;9: 262. doi:10.3390/biomedicines9030262
10. Alquwaizani M, Buckley L, Adams C, Fanikos J. Anticoagulants: A Review of the Pharmacology, Dosing, and Complications. Curr Emerg Hosp Med Rep. 2013;1: 83–97. doi:10.1007/s40138-013-0014-6
11. Ruigómez A, Schink T, Voss A, Herings RMC, Smits E, Swart-Polinder K, et al. Safety profile of rivaroxaban in first-time users treated for venous thromboembolism in four European countries. Fukumoto Y, editor. PLOS One. 2024;19: e0298596. doi:10.1371/journal.pone.0298596
12. Linkins L-A, Choi PT, Douketis JD. Clinical impact of bleeding in patients taking oral anticoagulant therapy for venous thromboembolism: a meta-analysis. Ann Intern Med. 2003;139: 893. doi:10.7326/0003-4819-139-11-200312020-00007
13. Witt DM, Clark NP, Kaatz S, Schnurr T, Ansell JE. Guidance for the practical management of warfarin therapy in the treatment of venous thromboembolism. J Thromb Thrombolysis. 2016;41: 187–205. doi:10.1007/s11239-015-1319-y
14. Shields LBE, Fowler P, Siemens DM, Lorenz DJ, Wilson KC, Hester ST, et al. Standardized warfarin monitoring decreases adverse drug reactions. Bmc Fam Pract. 2019;20: 151. doi:10.1186/s12875-019-1041-5
15. Burnett AE, Mahan CE, Vazquez SR, Oertel LB, Garcia DA, Ansell J. Guidance for the practical management of the direct oral anticoagulants (DOACs) in VTE treatment. J Thromb Thrombolysis. 2016;41: 206–232. doi:10.1007/s11239-015-1310-7
16. EINSTEIN Investigators, Rupert Bauersachs, Scott D Berkowitz, Benjamin Brenner, Harry R Buller, Hervé Decousus, et al. Oral rivaroxaban for symptomatic venous thromboembolism. N Engl J Med. 2010;363: 2499–2510. doi:10.1056/NEJMoa1007903
17. Wettermark B, Elseviers M, Almarsdóttir AB, Andersen M, Benko R, Bennie M, et al.

Introduction to drug utilization research. Drug Utilization Research. John Wiley & Sons, Ltd; 2016. pp. 1–12. doi:10.1002/9781118949740.ch1

18. Teng L, Xin H, Blix HS, Tsutani K. Review of the use of defined daily dose concept in drug utilisation research in China. Pharmacoepidem Dr S. 2012;21: 1118–1124. doi:10.1002/pds.3240
19. Keeling D, Baglin T, Tait C, Watson H, Perry D, Baglin C, et al. Guidelines on oral anticoagulation with warfarin - fourth edition. Br J Haematol. 2011;154: 311–324. doi:10.1111/j.1365-2141.2011.08753.x
20. White RH, Gettner S, Newman JM, Trauner KB, Romano PS. Predictors of rehospitalization for symptomatic venous thromboembolism after total hip arthroplasty. N Engl J Med. 2000;343: 1758–1764. doi:10.1056/NEJM200012143432403
21. Vaitkus P, Leizorovicz A, Cohen A, Turpie A, Olsson C-G, Goldhaber S, et al. Mortality rates and risk factors for asymptomatic deep vein thrombosis in medical patients. Thromb Haemost. 2005;93: 76–79. doi:10.1160/TH04-05-0323
22. Kucher N, Koo S, Quiroz R, Cooper JM, Paterno MD, Soukonnikov B, et al. Electronic alerts to prevent venous thromboembolism among hospitalized patients. N Engl J Med. 2005;352: 969–977. doi:10.1056/NEJMoa041533
23. Cohen AT, Agnelli G, Anderson FA, Arcelus JI, Bergqvist D, Brecht JG, et al. Venous thromboembolism (VTE) in Europe. The number of VTE events and associated morbidity and mortality. Thromb Haemost. 2007;98: 756–764.
24. Speth J. Guidelines in Practice: Prevention of Venous Thromboembolism. AORN J. 2023;118: 321–328. doi:10.1002/aorn.14019
25. Nicolaides AN, Fareed J, Kakkar AK, Comerota AJ, Goldhaber SZ, Hull R, et al. Prevention and treatment of venous thromboembolism--International Consensus Statement. Int Angiol: J Int Union Angiol. 2013;32: 111–260.
26. Braithwaite I, Healy B, Cameron L, Weatherall M, Beasley R. Lower limb immobilisation and venous thromboembolism risk: combined case-control studies. Postgrad Med J. 2017;93: 354–359. doi:10.1136/postgradmedj-2016-134365
27. Crainie CA, Cooper JG, Horner D. Thromboprophylaxis in ambulatory emergency department patients managed with lower limb immobilisation after injury: a national survey. Emerg Med J. 2023;40: 67–68. doi:10.1136/emermed-2022-212688
28. Fan P, Gao Y, Zheng M, Xu T, Schoenhagen P, Jin Z. Recent progress and market analysis of anticoagulant drugs. J Thorac Dis. 2018;10: 2011–2025. doi:10.21037/jtd.2018.03.95
29. Stevens SM, Woller SC, Kreuziger LB, Bounameaux H, Doerschug K, Geersing G-J, et al. Antithrombotic Therapy for VTE Disease: Second Update of the CHEST Guideline and Expert Panel Report. Chest. 2021;160: e545–e608. doi:10.1016/j.chest.2021.07.055
30. Wang L, Yao W. A Cohort Study on the Safety and Efficacy of Warfarin and Rivaroxaban in Anticoagulant Therapy in Patients with Atrial Fibrillation Study. Tang M, editor. Biomed Res Int. 2022;2022: 1–9. doi:10.1155/2022/4611383
31. Treceño-Lobato C, Jiménez-Serranía M-I, Martínez-García R, Corzo-Delibes F, Martín Arias L. New Anticoagulant Agents: Incidence of Adverse Drug Reactions and New Signals Thereof. Semin Thromb Hemost. 2019;45: 196–204. doi:10.1055/s-0038-1657783
32. Pham A, Heib A, Goodman E, Cotto R, Jafari P, Lipsitz E, et al. Warfarin versus direct oral anticoagulants for patients needing distal deep vein thrombosis treatment. J Vasc Surg: Venous

Lymphat Disord. 2022;10: 826-831.e1. doi:10.1016/j.jvsv.2022.01.006

33. Lawal OD, Aronow HD, Hume AL, Shobayo F, Matson KL, Barbour M, et al. Venous thromboembolism, chronic liver disease and anticoagulant choice: effectiveness and safety of direct oral anticoagulants versus warfarin. Res Pract Thromb Haemost. 2024;8: 102293. doi:10.1016/j.rpth.2023.102293
34. McHorney CA, Ashton V, Laliberté F, Germain G, Wynant W, Crivera C, et al. Adherence to Rivaroxaban Compared with Other Oral Anticoagulant Agents Among Patients with Nonvalvular Atrial Fibrillation. J Manag Care Spec Pharm. 2017;23: 980–988. doi:10.18553/jmcp.2017.23.9.980
35. Yeh CH, Hogg K, Weitz JI. Overview of the new oral anticoagulants: opportunities and challenges. Arterioscler, Thromb, Vasc Biol. 2015;35: 1056–1065. doi:10.1161/ATVBAHA.115.303397
36. De Caterina R, Renda G, Carnicelli AP, Nordio F, Trevisan M, Mercuri MF, et al. Valvular Heart Disease Patients on Edoxaban or Warfarin in the ENGAGE AF-TIMI 48 Trial. J Am Coll Cardiol. 2017;69: 1372–1382. doi:10.1016/j.jacc.2016.12.031
37. Ezekowitz MD, Nagarakanti R, Noack H, Brueckmann M, Litherland C, Jacobs M, et al. Comparison of Dabigatran and Warfarin in Patients With Atrial Fibrillation and Valvular Heart Disease: The RE-LY Trial (Randomized Evaluation of Long-Term Anticoagulant Therapy). Circulation. 2016;134: 589–598. doi:10.1161/CIRCULATIONAHA.115.020950
38. Otto CM, Nishimura RA, Bonow RO, Carabello BA, Erwin JP, Gentile F, et al. 2020 ACC/AHA Guideline for the Management of Patients With Valvular Heart Disease: Executive Summary: A Report of the American College of Cardiology/American Heart Association Joint Committee on Clinical Practice Guidelines. Circulation. 2021;143: e35–e71. doi:10.1161/CIR.0000000000000932
39. Liu Z, Luo F, Zhao J, Chen W, Gao W, Zhou Z. Association between gene polymorphisms and initial warfarin therapy in patients after heart valve surgery. Pharmacol Rep. 2024;76: 390–399. doi:10.1007/s43440-024-00575-8
40. Kusumoto FM, Calkins H, Boehmer J, Buxton AE, Chung MK, Gold MR, et al. HRS/ACC/AHA expert consensus statement on the use of implantable cardioverter-defibrillator therapy in patients who are not included or not well represented in clinical trials. Heart Rhythm. 2014;11: 1270–1303. doi:10.1016/j.hrthm.2014.03.041
41. Feng K, Wang K, Zhou Y, Xue H, Wang F, Jin H, et al. Non-Anticoagulant Activities of Low Molecular Weight Heparins-A Review. Pharmaceuticals. 2023;16: 1254. doi:10.3390/ph16091254
42. Chu H, Hu B, Huang X, Chai Y, Zhou D, Wang Y, et al. Host and viral determinants for efficient SARS-CoV-2 infection of the human lung. Nat Commun. 2021;12: 134. doi:10.1038/s41467-020-20457-w
43. Islam M. Sodium bicarbonate is a potential alternative to classic heparin as a lock solution for non-tunneled dialysis catheters: a result from the prospective randomized BicarbLock trial. Int Urol Nephrol. 2023;56: 1465–1474. doi:10.1007/s11255-023-03821-9
44. Bender F, Aronson L, Hougie C, Moser K. Bioequivalence of subcutaneous calcium and sodium heparins. Clin Pharmacol Ther. 1980;27: 224–229. doi:10.1038/clpt.1980.35
45. Junqueira DR, Zorzela LM, Perini E. Unfractionated heparin versus low molecular weight heparins for avoiding heparin-induced thrombocytopenia in postoperative patients. Cochrane

Vascular Group, editor. Cochrane Db Syst Rev. 2017;2017: CD007557. doi:10.1002/14651858.CD007557.pub3
46. Hirsh J, De Vries TAC, Eikelboom JW, Bhagirath V, Chan NC. Clinical Studies with Anticoagulants that Have Changed Clinical Practice. Semin Thromb Hemost. 2023;49: 242–254. doi:10.1055/s-0042-1760330
47. Long H, Yang Y, Geng X, Mao Z, Mao Z. Changing Characteristics of Pharmaceutical Prices in China Under Centralized Procurement Policy: A Multi-Intervention Interrupted Time Series. Front Pharmacol. 2022;13: 944540. doi:10.3389/fphar.2022.944540
48. Zhang Q, Wang R, Chen L, Chen W. Effect of China national centralized drug procurement policy on anticoagulation selection and hemorrhage events in patients with AF in Suining. Front Pharmacol. 2024;15: 1365142. doi:10.3389/fphar.2024.1365142

Figure 1

Click here to access/download;Figure;Figure 1.tiff

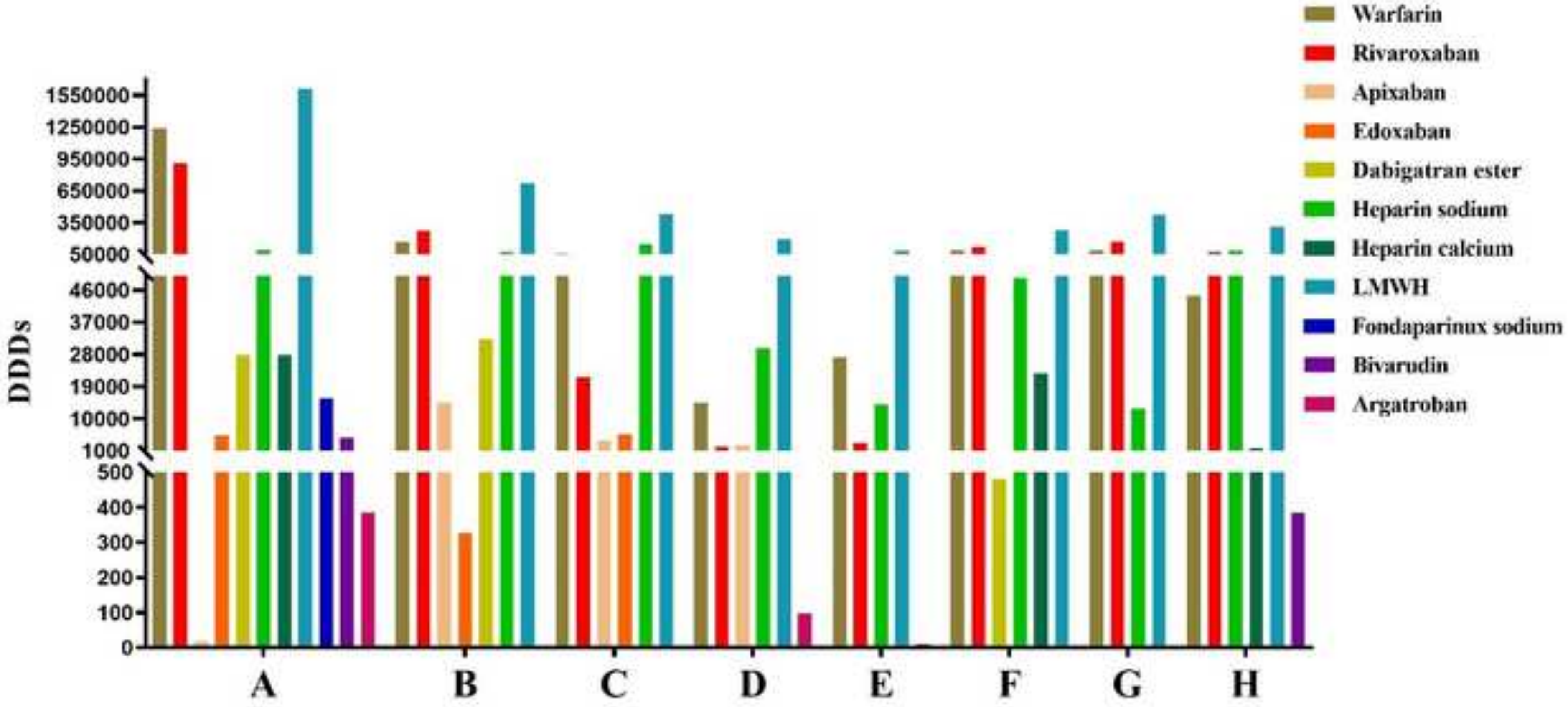

Figure 2

Click here to access/download;Figure;Figure 2.tiff

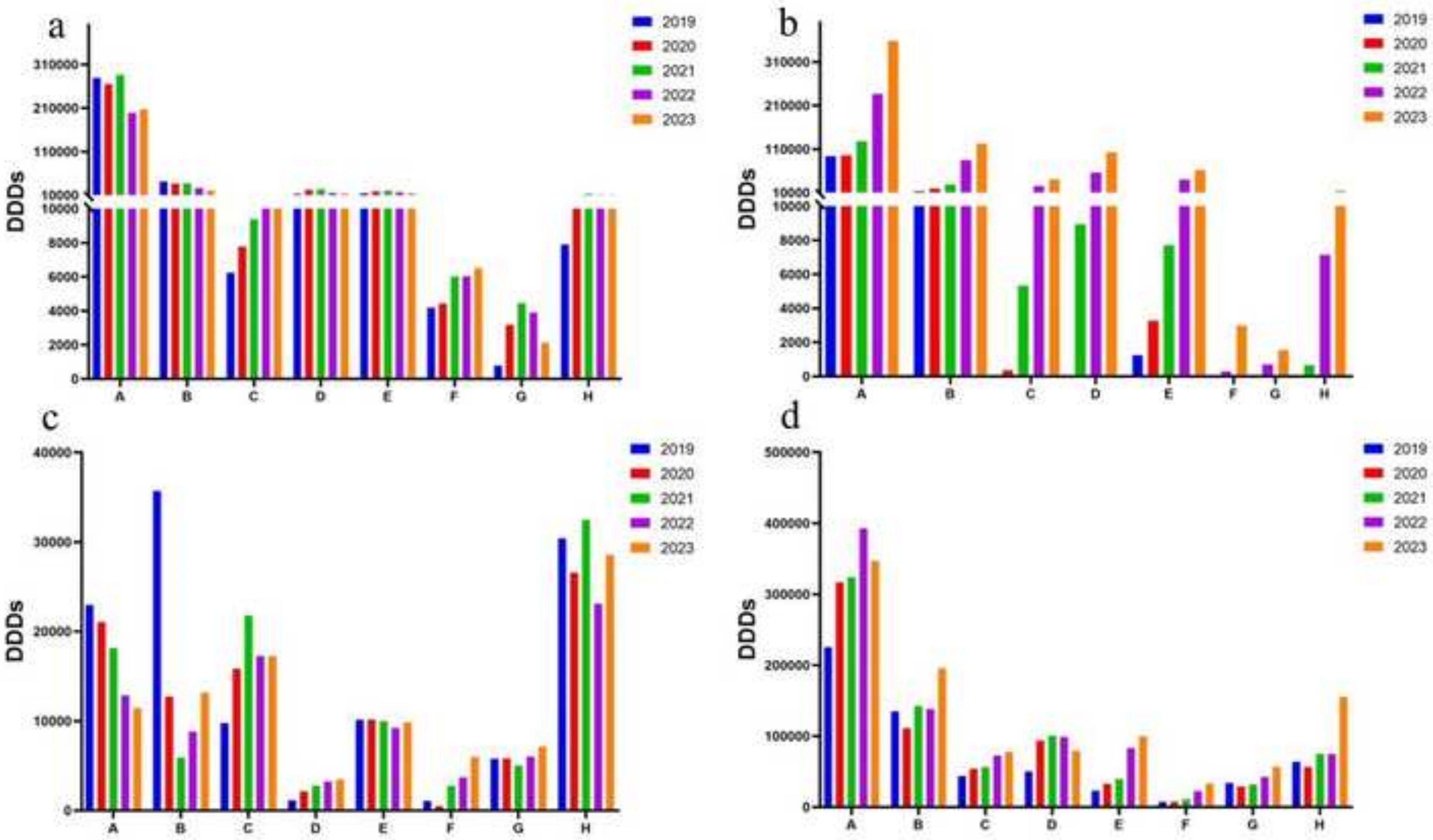

Figure 3

Click here to access/download;Figure;Figure 3.tiff

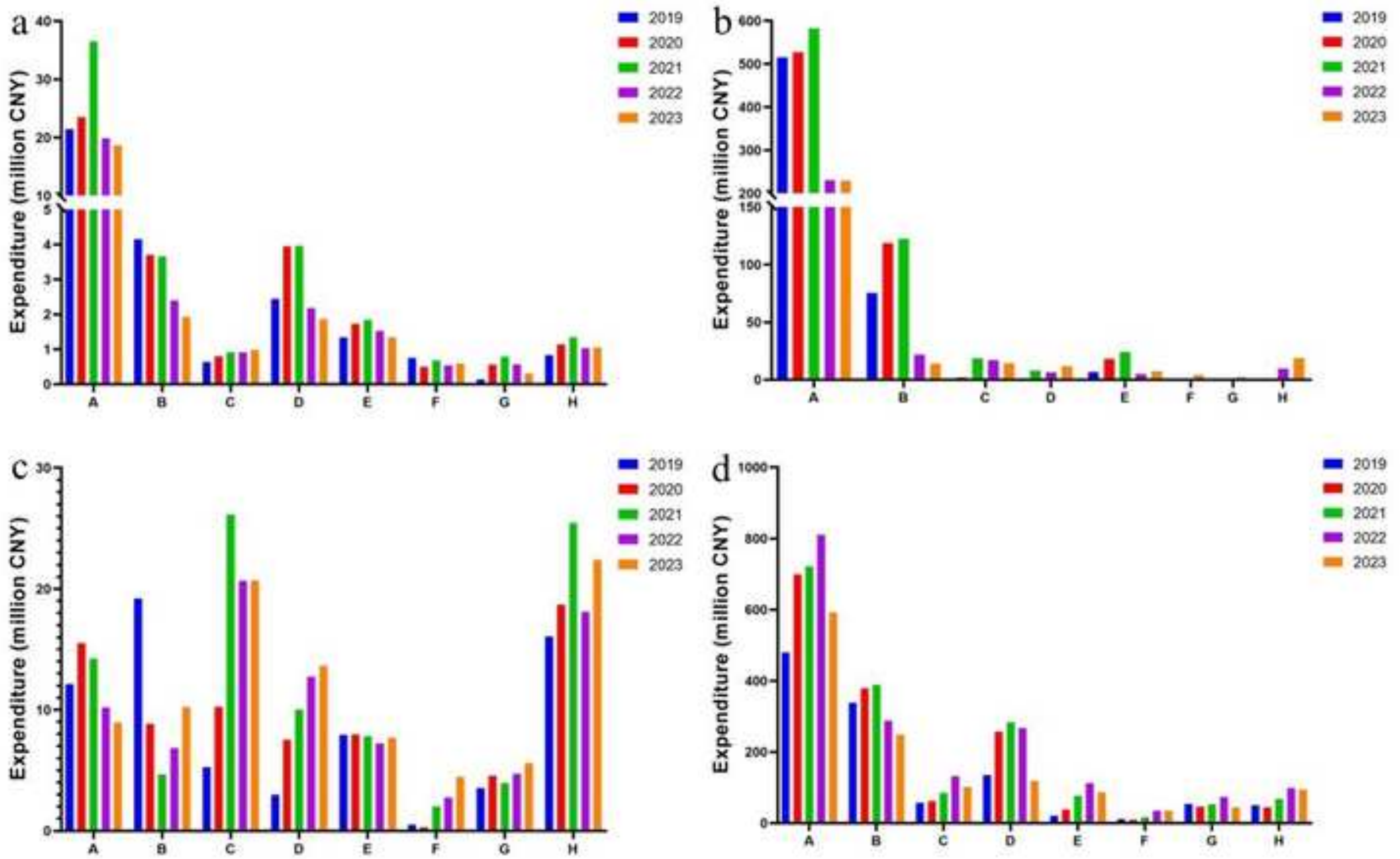

Figure 4

Click here to access/download;Figure;Figure 4.tiff

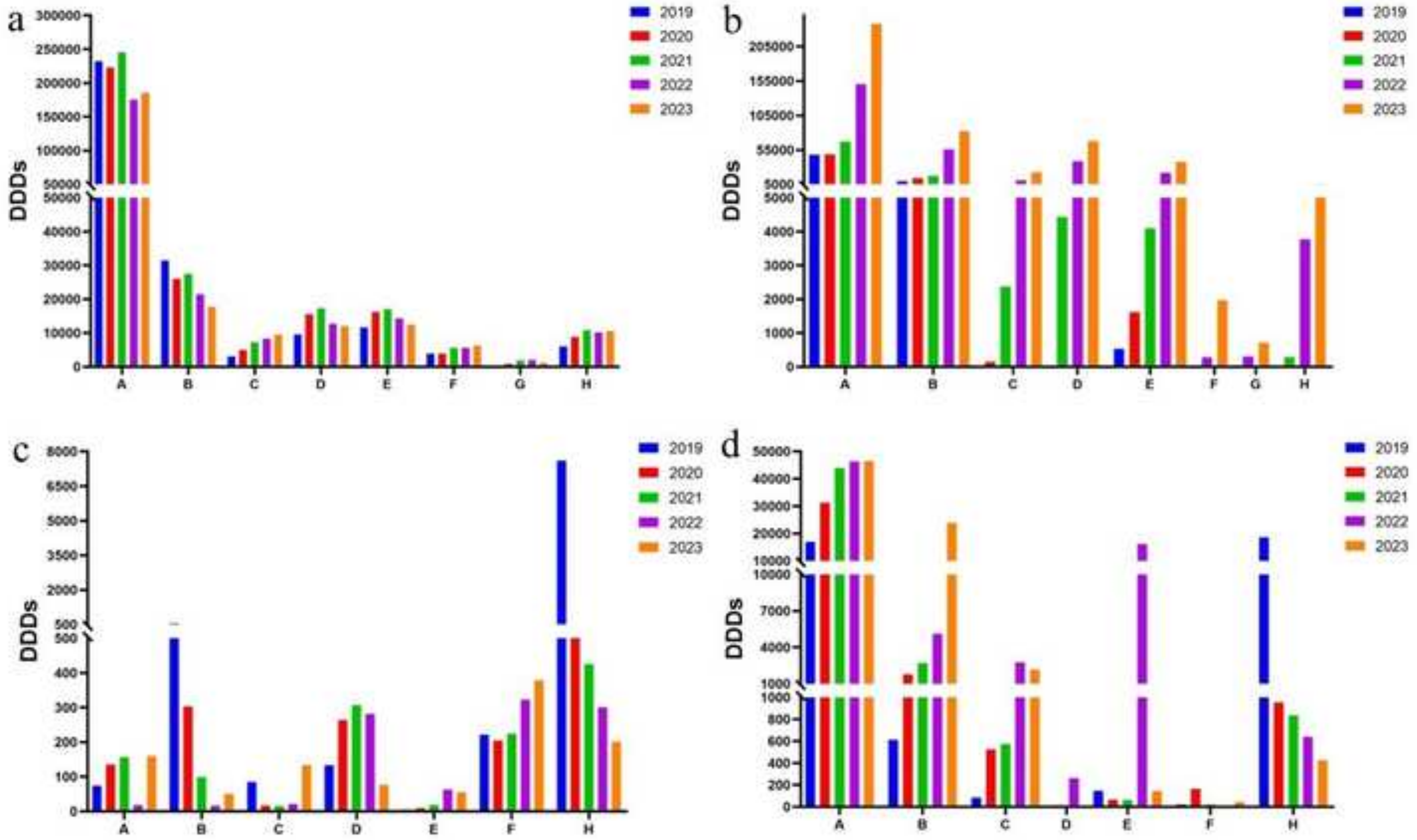

Figure 5

Click here to access/download;Figure;Figure 5.tiff

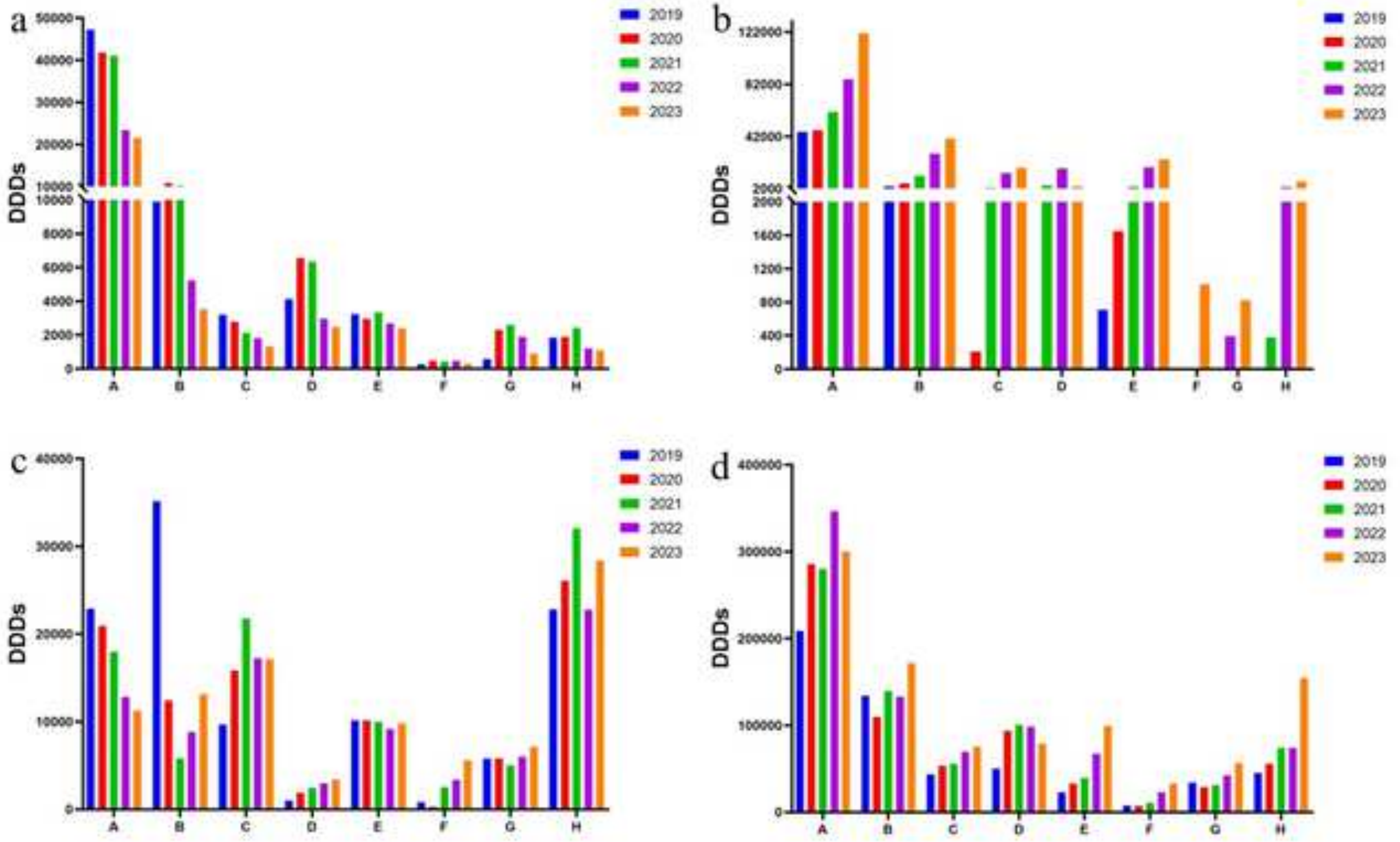

Figure 6

Click here to access/download;Figure;Figure 6.tiff

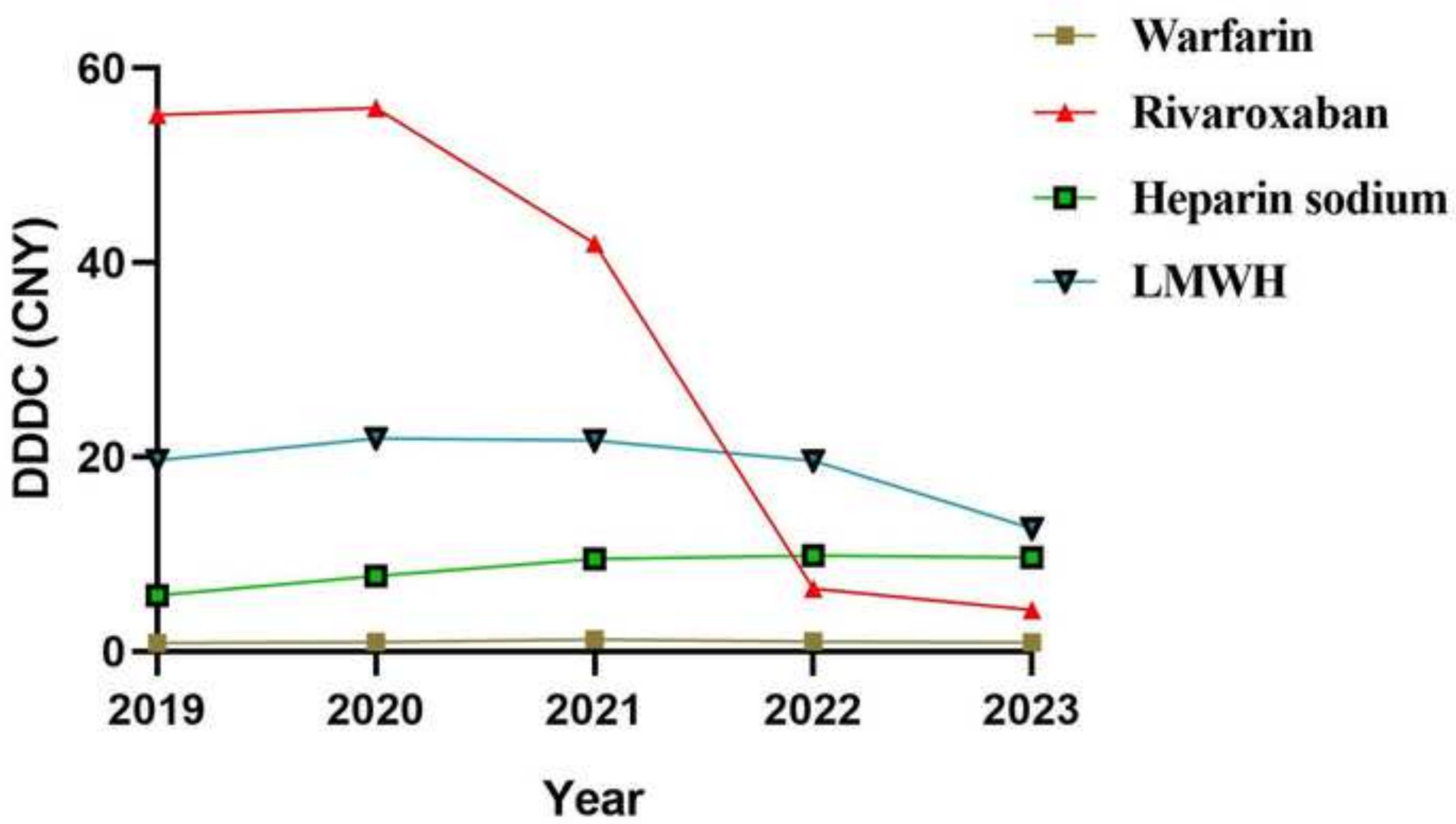

Supplement: Attachment — Submitted filename: PONE-D-24-36242_reviewer.pdf [file pone.0318463.s006.pdf]
