# Supplementary material for: Anticoagulants utilization in eight hospitals within the Luzhou region from 2019 to 2023
Source: PLoS One. 2025 Jan 31;20(1):e0318463. doi: 10.1371/journal.pone.0318463 (PMC11785323; doi:10.1371/journal.pone.0318463)
Supplement: Attachment — Submitted filename: Alyaa Ajaboor (review for PLOS).docx [file pone.0318463.s005.docx]

This study presented a descriptive analysis of anticoagulants use over 4 years duration in 8 hospitals in Luzhou region in China. The main idea of this study appears clear, but the poor quality of writing could make it challenging for readers to comprehend. I recommend that this study undergo **Major** editorial and language revisions to enhance clarity and readability.

These are my comments:

**Study title**

I advise to change the title to: anticoagulants utilization in eight hospitals within the Luzhou region: descriptive study from 2019 to 2023

**Abstract**

Line 18 to 20: Rephrase the background of the abstract. It’s not supposed to state the objective but rather to highlight the gap in the literature and briefly mention the aim of the study

Line 22 to 23: “Data on the utilization of anticoagulant drugs in tertiary hospital within a district from 2019 to 2023 were collected” this sentence needs rewording. It’s clearer to say tertiary-care hospitals throughout.

Line 24: “Diagrams illustrating the trend of change were generated”. Change in what? Either clarify the sentence or remove it if it’s not related to the main analysis of this study

Line 28: Define CNY on first mention in the abstract.

Line 33 to 36: This is not a proper conclusion. Suddenly VTE was mentioned!! is it something that you estimated in this study? (e.g., Prevalence of VTE) how did you assume that the VTE rate is increasing? I advise rewriting the conclusion to provide a proper summary of the study findings and its clinical impact.

**Background**

Line 48: “as same as”?!!

Line 49: “1-2 per”??!!

Line 52: “four consecutive years” clarify and provide reference.

Line 60 to 61: “VTE in the course of the disease is usually divided into three different stages” I advise to rephrase this sentence.

Line 67: “ a clear index” not clear what do you mean here!! Do you mean clear indication? Clarify please.

Line 67 to 69: “drug treatment mechanism (hepatic and renal metabolism), variable drug half-life, treatment requirements, monitoring needs” these factors sound a bit confusing and could overlap in their meanings. Treatment mechanism, you could just say mechanism of actions. Hepatic and renal metabolism as well as half-life could all go under pharmacokinetics. Not sure what you mean by treatment requirements!!!! You could just remove it and say therapeutic drug monitoring.

Line 82: “With the publication of “Good Manufacturing Practice” provide a reference here.

Line 85 to 92: there seems to be too much describing of DUR here. Defining what DDD or DDDC should be in the methods section and should not take this much space from the study introduction or background. This part needs rephrasing to highlight the main gaps in the literature that the study will address.

Line 93 to 95: “This study aims to luzhou area through the analysis of the drug utilization research in tertiary hospital nearly five years of anticoagulant drugs usage, combining economics discussion and thinking of anticoagulant drugs selection and reasonable use.”

I advise rephrase this part and make sure its grammatically sound.

**Methods**

Overall, the methods need major structural revision. The subheading needs to be divided as follows: Data source, Data collection, then statistical analysis.

Line 97: the subheading “Data collection” needs to be changed as I mentioned earlier and this section should be dedicated to explaining the *data sources* such as explaining the data system of the eight hospitals included in this study and if there is any coding system used to extract the necessary data.

Line 99: “data collection began in March 2024”, this does not sound correct. If it’s a retrospective study and the study observational window is from 2019 to 2023.

Line 100 to 101: “Third-Level Grade A (A, B) and Third-Level Grade B (C, D, E, F, G, H)” explain to the reader what grading this is and provide references.

Line 101 to 106: all this part needs to be transferred to the data collection section.

Line 102: “medication specifications” what does this mean exactly?

Line 103: “quantities and amounts” clarify the difference.

Line 103: why saying “etc.”? I believed there are five approved DOACs its better to mention all of them

Line 105: same as previous comment

Line 110: here its better to change the subheading to *Data collection* and add the description of the WHO definition of DDD and DDDC.

Line 111: create a new subheading for the statistical analysis and describe in detail how you summarized the data using descriptive statistics. Therefore remove “descriptive statistics” from this part.

Line 120 to 121: “according to the management scheme suggested in the manufacturer's instructions approved by the Food and Drug Administration” not clear its better to provide an example.

Liner 129: “China expert consensus of warfarin anticoagulant therapy”, "Guidelines on

oral anticoagulation with warfarin – fourth edition" provide reference

Line 132 to 135: provide here a proper statistical analysis section and describe in details the summary measures used in describing the results.

**Results**

Same as in methods the subheadings used needs modification “Different hospital drug use” is not an appropriate nor “Overall trends in anticoagulants” or “The difference of anticoagulant drugs outpatient and inpatient”. It might be better to change these subheadings to “Utilization and Trends of Anticoagulant Drugs” another for “Expenditure of Anticoagulant Drug Utilization” and another one for “Outpatient vs. Inpatient Utilization of Anticoagulant” then adjust the text accordingly.

Line 138 to 147: this whole paragraph needs rewriting to provide a numeric description of the main results around the utilization trends with reference to Figure 1.

Figure 1 it’s not clear these results are for which year or if its for the whole observation period. Clarify please.

Line 149 to 150: “The prevalence of VTE patients has been on the rise, consequently elevating the significance of anticoagulant medications” this statement is not supposed to be included here. If you think that the rise in anticoagulants utilization is related to the rise in the prevalence of VTE, you should address this in the discussion providing the appropriate references to support your claims. However, since you have not estimated the prevalence of VTE in this study then it should not be included in the results.

Line 175: “In the past five years, there has been a noticeable upward trend in the number of outpatient visits and inpatient” is something you have measured in this study (number of outpatient visits)?!!! Clarify please. If not, then just simply say between the year 20xx and 20xx ………and described anticoagulants utilization in the outpatient settings.

Line 182 to 183: “This finding also indicates the prevailing utilization of oral anticoagulants as the primary choice for outpatient anticoagulation therapy” this more suitable to mention in discussion.

Line 187: “significant margin” you should be careful when you use the word significant as its commonly used to describe any significant statistical differences interpreted from P values or confidence intervales. Since no statistical tests were performed in this study and was only done using descriptive statistics, then its not appropriate to use this phrase.

Line 197 to 202: this whole paragraph is not suitable for results. Better to remove or include in discussion.

Table 1: it’s not clear why this table is only for warfarin? and which year is it describing? It would be clearer to include all anticoagulants and describe each year separately (for both out- and inpatient utilization).

**Discussion**

The first paragraph in the discussion needs to highlight the design and purpose of this study and description in words of the main findings.

Line 227 to 235: I advise removing this part and link line 224 to 226 with the paragraph in line 236.

Line 236 to 240: need a bit of clarifying so the reading know that you are referring to your study findings.

Line 257 to Line 259: again, clarify that you are referring to your study findings. Discussion is not supposed to include any reference for figures or tables.

Line 270 and 290: these two paragraphs need to be linked to the findings of your study.

Line 312: “This is the reason” In observational studies it’s better to avoid definitive statements, particularly in a descriptive study like this one as you have not study any associations and you are only describing what is happening in real life settings.

Line 328 to 331: “Furthermore, our assessment solely focused on the appropriateness of warfarin prescription for its specific indication, without considering the suitability of prescribing alternative anticoagulants or the practices followed by other medical institutions in terms of anticoagulant prescriptions”. I don’t believe you have studied the appropriateness of warfarin prescribing, rather you have only mentioned the indications. Which is not enough to assess the appropriateness of prescribing, that requires extracting clinical data at the patient-level.

Line 338 to 339: “owing to their minimal adverse reactions and remarkable therapeutic efficacy” again careful with definitive statements.
