# Supplementary material for: Anticoagulants utilization in eight hospitals within the Luzhou region from 2019 to 2023
Source: PLoS One. 2025 Jan 31;20(1):e0318463. doi: 10.1371/journal.pone.0318463 (PMC11785323; doi:10.1371/journal.pone.0318463)
Supplement: Attachment — Submitted filename: Response to Reviewers.docx [file pone.0318463.s007.docx]

**Response to Reviewers**

**Reviewer #1** **Comments to the Author:** This article provides a valuable and timely analysis of anticoagulant drug utilization trends across multiple tertiary hospitals in the Luzhou region from 2019 to 2023.
However, to strengthen the statistical rigor and enhance the interpretability of the results, I assert necessity to including inferential statistical tests where comparisons are made across groups (e.g., DDD trends, cost differences). Including appropriate inferential tests would allow the authors to draw more robust conclusions regarding observed trends and differences, enhancing the article's scientific validity.
Ethically, the study appears to be conducted with attention to patient privacy, using anonymized data, and the authors have appropriately disclosed the lack of conflicts of interest. I did not observe any issues with dual publication or ethical concerns that would compromise the integrity of the research.
Overall, this study is well-structured, addresses an important healthcare issue, and has the potential to contribute valuable insights to the field of anticoagulation therapy. With the addition of inferential statistics and some clarification in specific sections, this work would represent a strong and impactful addition to the literature.

Reply: Thank you for your valuable scientific comments and endorsement of our manuscript. With your help, we have added inferential statistical tests to enhance statistical rigor and improve the interpretability of the results. Thank you very much for your valuable suggestions on our manuscript.

**Comment 1.** Line 19 to 20: “and provide valuable insights for their management and rational use”. Please clarify how this study contributes to existing literature on anticoagulant use in hospital settings.

Reply: Thank you for your invaluable advice. We have revised the background of the abstract to explicitly highlight the implications of our article on anticoagulant use in various healthcare settings. The revised background (line:24-27) is as follows:

With the increasing utilization of anticoagulants, the selection of appropriate anticoagulants has emerged as a significant quandary. The objective of this study was to evaluate recent trend in the utilization and expenditure of anticoagulants within a specific region, aiming to provide valuable insights into the optimal choice of anticoagulants across other healthcare facilities.

**Comment 2.** Line 27: “The DDDC of rivaroxaban decreased”. Adding explanation of this unexpected findings would enlighten reader.

Reply: Thank you for your invaluable advice. We have revised the results to provide a comprehensive explanation of the factors contributing to the decrease in DDC, aiming to enhance readers' comprehension of the article. The revised version (line: 36-41) is as follows:

The implementation of the centralized procurement policy, however, resulted in a decline in the expenditure of rivaroxaban and LMWH in 2021 and 2022 respectively. The DDC value of rivaroxaban experienced a substantial decrease over the past five years (*p* = 0.020), declining from 55.20 Chinese Yuan (CNY) in 2019 to 4.28 CNY in 2023. Conversely, there was a slight increase noted in the DDC of heparin sodium during this time frame (*p* = 0.042).

**Comment 3.** Line 28: “significantly”. Clarify any statistical tests used for comparing DDDC trends across hospitals, as this is not mentioned. **Significance** cannot be assessed using only descriptive statistics.

Reply: Thank you for your valuable scientific comment. We sincerely apologize for the oversight that led to an error in this section. In order to address this issue, we have added linear regression analysis to the article in order to show the trend of defined daily doses (DDDs) and defined daily cost (DDC), which helps strengthen the scientific rigor of our study. For example (line:242-246):

The DDC value of rivaroxaban exhibited a significant decrease over the past 5 years (*p* = 0.020), declining from 55.20 CNY in 2019 to 4.28 CNY in 2023. Furthermore, there was a slight increase observed in the DDC of heparin sodium during the same period (*p* = 0.042), rising from 5.79 CNY in 2019 to 9.67 CNY in 2023. However, the DDC values of warfarin (*p* = 0.793) and LMWH (*p* = 0.202) remained relatively stable throughout the past 5 years.

**Comment 4.** Line 33-36. Conclusion did not reflect on the main results of the study. I think, your conclusion should more directly reflect the study’s key results, highlighting the observed trends in anticoagulant usage, etc. Especially highlighting and explaining the discrepancy found between DDDs and DDDC.

Reply: Thank you for your valuable scientific comment. The conclusions in the abstract have been revised to more accurately reflect the key findings of the study, with the revised conclusions (line: 42-45) presented as follows:

Over the past five years (2019-2023), there has been an increase in the utilization of rivaroxaban and LMWH. However, their expenditure has decreased. In addition, the utilization and expenditure of warfarin and heparin sodium remained relatively stable. The application prospects of rivaroxaban and LMWH are promising.

**Comment 5.** Line 93-95. It’s helpful to highlight gaps this study fills regarding regional drug utilization. Consider suggesting more details on these gaps for the reader’s understanding.
Also, please clarify the significance of selecting the Luzhou region and tertiary hospitals for this study.

Reply: Thank you for your valuable advice. We have incorporated the rationales and significance behind the selection of tertiary-care hospitals in Luzhou, and the specific modifications (line:113-121) as follows:

The city of Luzhou, situated in the southern region of Sichuan province, stands as a representative example with relatively high medical and economic standards within the province. Moreover, owing to factors such as advanced medical facilities and convenient transportation, tertiary-care hospitals are predominantly chosen by patients for their treatment needs. Consequently, this inclination towards tertiary-care hospitals results in a greater utilization of anticoagulant medications and facilitates a more comprehensive understanding of their usage trends. Therefore, the objective of this study is to provide an all-encompassing overview regarding the utilization and expenditure patterns of anticoagulant drugs in Luzhou's tertiary-care hospitals from 2019 to 2023 through DUR.

**Comment 6.** Line 140: “luzhou”. Please Capitalize Luzhou wherever mentioned.

Reply: We agree with you and apologize for our mistake. We have revised it. Thank you for your positive and progressive suggestions on our manuscript.

**Comment 7.** Line 227: “An estimated 370,000 deaths related to VTE occur annually in six European countries”. Is there any estimation in China?

Reply: Thank you for your valuable advice. We removed the VTE example from Europe and added the epidemiology of VTE in China, with the following changes (line:253-255):

In China, the hospitalization crude rate of VTE was 2.9 in 2007 to 15.8 in 2016 per 100,000 population, with a relative increase of five-fold (*p* < 0.001)[1].

**Reference**

[1].Zhang Z, Lei J, Shao X, Dong F, Wang J, Wang D, et al. Trends in Hospitalization and In-Hospital Mortality From VTE, 2007 to 2016, in China. Chest. 2019;155: 342–353. doi:10.1016/j.chest.2018.10.040

**Comment 8.** Line 228-235. These sentences present general information on VTE prevention and treatment, which does not directly explain or support your study’s findings. Consider moving it to the introduction if you feel it provides necessary background context. In the discussion, focus on interpreting your results in relation to existing research and highlighting specific insights drawn from your data.

Reply: Thank you very much for your scientific comment. We omitted general information about VTE prevention and treatment and focused on explaining results relevant to existing research and highlighting specific insights. The specific changes are as follows (lines 255-259):

Therefore, the utilization of anticoagulants is of utmost importance. The findings from this study demonstrate a notable increase in the utilization of rivaroxaban and LMWH over the past 5 years, accompanied by a decrease in expenditure due to changes in DDC. Simultaneously, their exceptional safety profile and high efficacy distinguish them as superior options among various anticoagulants.

**Comment 9.** Line 241-242. What does “nationwide” mean in this context? This is confusing because apparently these two studies were not conducted in China. Please change the sentence to remove that ambiguity.

Reply: We agree with you, and we apologize for this ambiguous sentence. The specific changes are as follows (line:265-267):

In a UK national survey and a randomized, controlled trial, outpatient prophylaxis with LMWH demonstrated efficacy in mitigating the risk of VTE among patients with immobilization of the lower extremities.

**Comment 10.** Line 265-267. The term 'AD' is unclear and should be defined for clarity, at least in the first appearance. Additionally, the inclusion of P-values and using the technical term “significant”, suggest inferential statistical analysis, yet your methodology states that only descriptive statistics were used. Please clarify if inferential tests were conducted and, if so, detail these methods in the methodology section. If not, consider removing the P-values and “significant(ly)” terms; to maintain consistency with your stated analysis approach.

Reply: Thank you very much for your scientific comment. We have redefined 'AD' to make its meaning clearer to the reader. In addition, we added inferential statistical methods and included *p* values.

**Comment 11.** Line 270-273. Again, as above. These are general knowledge, and suggested to be moved into background section if necessary.

Reply: Thank you for your valuable advice. We also agree with you and have deleted most of the content. However, considering that the discussion in this section is mainly to analyze the specific indications for warfarin in order to determine the rationality of its prescription. We also retained some of the necessary content. The specific changes are as follows (line: 295-303):

Warfarin is the most widely utilized oral anticoagulant globally for the prevention and treatment of various thrombotic events, including deep-vein thrombosis, atrial fibrillation, valvular heart disease and prosthetic valves, as well as peripheral vascular disease. However, according to the expert consensus statement on ICD therapy, administration of warfarin for anticoagulation purposes is not recommended following ICD surgery. Therefore, the use of warfarin after ICD implantation in our study was not justified; however, it constitutes a negligible proportion. Despite its flaws, the majority of warfarin diagnoses in this study were in accordance with the prescribed indications for warfarin. However, collaborative efforts are still required to further enhance the rationality of prescribing practices.

**Comment 12.** Line 308-309. Is there any expected effect of COVID-19 on this trend?

Reply: Reply: Thank you for your valuable scientific advice. Patients with COVID-19 have a certain risk of thrombosis, and the guidelines require that patients with COVID-19 need to use heparin drugs for anticoagulant therapy [2]. In addition, the duration of COVID-19 was from December 2019 to December 2022, which was the window period of our study. Thus, COVID-19 had an effect on trends in the use of heparin.

**Reference**

[2]. National Health Commission of the People’s Republic of China. Diagnosis and Treatment of COVID-19(Trial Version 10). Chinese Journal of Clinical Infectious Diseases. 2023;16(1):1-9. Doi:10.3760/cma.j.issn.1674-2397.2023.01.001

**Comment 13.** Line 319-334. Limitations have been addressed very well. However, it may be useful to recommend more specific future research directions, such as including secondary hospitals or exploring anticoagulant trends by patient demographics (age, comorbidities).

Reply: Thank you for your valuable advice and affirmation. We have added more specific future research directions with the following modifications (line:352-354):

In future studies, we will further investigate the adaptability of other anticoagulants in prescription and consider their inclusion in secondary hospitals or continue to explore trends in anticoagulants based on patient demographic characteristics such as age and comorbidities.

**Comment 14.** Line 339-340:” The implementation of the centralized procurement policy has significantly reduced the prices of anticoagulant drugs”. Good point. Please point to it briefly in the abstract.

Reply: Thank you for your valuable advice and affirmation. We have pointed out in the abstract. Thank you for your positive and progressive suggestions on our manuscript.

**Reviewer #2** **Comments to the Author:** This study presented a descriptive analysis of anticoagulants use over 4 years duration in 8 hospitals in Luzhou region in China. The main idea of this study appears clear, but the poor quality of writing could make it challenging for readers to comprehend. I recommend that this study undergo Major editorial and language revisions to enhance clarity and readability.
Find in the attached my comments.

Reply: Thank you very much for your scientific comment. We apologize for grammatical errors and poor writing quality. With your help, we made significant language changes to the manuscript to improve the readability of the article. Thank you for your positive suggestions on our manuscript.

**Comment 1.** Line 1 to 2. I advise to change the title to: anticoagulants utilization in eight hospitals within the Luzhou region: descriptive study from 2019 to 2023.

Reply: Thank you for your valuable advice. Integrating the opinions of multiple reviewers, the full text adds inferential statistical analysis on the basis of the first draft, which makes the article more convincing and rigorous. The title of this article is not suitable for descriptive analysis, but we have changed it accordingly (lines: 1-2):

Anticoagulants utilization in eight hospitals within the Luzhou region from 2019 to 2023.

**Comment 2.** Line 18 to 20: Rephrase the background of the abstract. It’s not supposed to state the objective but rather to highlight the gap in the literature and briefly mention the aim of the study

Reply: Thank you very much for your scientific comment. We modified the background section so that it was no longer a description of the research purpose only, and the revised background was as follows (line:24-27):

With the increasing utilization of anticoagulants, the selection of appropriate anticoagulants has emerged as a significant quandary. The objective of this study was to evaluate recent trend in the utilization and expenditure of anticoagulants within a specific region, aiming to provide valuable insights into the optimal choice of anticoagulants across other healthcare facilities.

**Comment 3.** Line 22 to 23: “Data on the utilization of anticoagulant drugs in tertiary hospital within a district from 2019 to 2023 were collected” this sentence needs rewording. It’s clearer to say tertiary-care hospitals throughout.

Reply: Thank you very much for your scientific comment. We modified the sentence to make it more colloquial. The specific changes are as follows (line:28-30):

The data on anticoagulant utilizations in tertiary-care hospitals within a district were collected from January 2019 to December 2023.

**Comment 4.** Line 24: “Diagrams illustrating the trend of change were generated”. Change in what? Either clarify the sentence or remove it if it’s not related to the main analysis of this study.

Reply: Thank you for your valuable advice. What we intended to describe was to plot trends in the frequency of use (DDDs), DDC, and cost of anticoagulants. We apologize for any ambiguity. We modified it as follows (lines 31-32):

The trends in the utilization and expenditure of anticoagulants were examined using linear regression analysis.

**Comment 5.** Line 28: Define CNY on first mention in the abstract.

Reply: We agree with you and have defined "CNY". Thank you for your positive suggestions on our manuscript.

**Comment 6.** Line 33 to 36: This is not a proper conclusion. Suddenly VTE was mentioned!! is it something that you estimated in this study? (e.g., Prevalence of VTE) how did you assume that the VTE rate is increasing? I advise rewriting the conclusion to provide a proper summary of the study findings and its clinical impact.

Reply: Thank you very much for your valuable scientific advice. VTE was not assessed in our study. VTE was introduced because the starting point of this study was to find an increase in VTE hospitalizations, which led to an increase in the use of anticoagulants. We then used VTE as a background for the manuscript. We modified the conclusions to make the results of this study more salient. Specific changes are as follows (42-45):

Over the past five years (2019-2023), there has been an increase in the utilization of rivaroxaban and LMWH. However, their expenditure has decreased. In addition, the utilization and expenditure of warfarin and heparin sodium remained relatively stable. The application prospects of rivaroxaban and LMWH are promising.

**Comment 7.** Line 48: “as same as”?!!

Reply: Thank you for your valuable advice. We deeply apologize. We would like to express that the incidence and mortality of VTE are comparable to those of myocardial infarction. We modified it as follows (line:71-73):

VTE affects millions of individuals globally each year and is associated with morbidity and mortality rates comparable to those observed in myocardial infarction.

**Comment 8.** Line 49: “1-2 per”??!!

Reply: Reply: Thank you for your valuable advice. We apologize for this mistake. What we want to express in the manuscript is that the estimated VTE incidence ranged from 1 per 1000 to 2 per 1000 population. We have revised it as follows (line:73-75):

In Europe and the United States, the estimated VTE incidence ranged from 1 per 1000 to 2 per 1000 population. Comparatively, Asia exhibits a lower rate of VTE compared to Europe and the United States.

**Comment 9.** Line 52: “four consecutive years” clarify and provide reference.

Reply: Thank you for your valuable advice. We revised it to clarify the specific year and add references. The specific changes are as follows (line:75-76):

Notably in China, VTE prevention has been listed as one of the top ten national goals for medical safety improvement for four consecutive years (2021-2024)[3–6].

**Reference**

[3].National Health Commission of the People’s Republic of China [Internet]. National healthcare quality and safety improvement targets in 2021. 2021 Feb 20 [cited 2024 Nov 19]. Available from: http://www.nhc.gov.cn/yzygj/s7657/202102/8c53313663284a7ba146648509538ee2.shtml

[4].National Health Commission of the People’s Republic of China [Internet]. National healthcare quality and safety improvement targets in 2022. 2022 Mar 2 [cited 2024 Nov 21]. Available from: http://www.nhc.gov.cn/yzygj/s3585/202203/ffed3474b1884058841a07c144ad094e.shtml

[5].National Health Commission of the People’s Republic of China [Internet]. National healthcare quality and safety improvement targets in 2023. 2023 Feb 28 [cited 2024 Nov 21]. Available from: http://www.nhc.gov.cn/yzygj/s7657/202302/a61fc382f3b64c7e99dafbf8cf4da8a1.shtml

[6].National Health Commission of the People’s Republic of China [Internet]. National healthcare quality and safety improvement targets in 2024. 2024 Feb 1 [cited 2024 Nov 21]. Available from: http://www.nhc.gov.cn/yzygj/s7657/202402/6aea7c6510da48a6b50e84417b4f30a3.shtml

**Comment 10.** Line 60 to 61: “VTE in the course of the disease is usually divided into three different stages” I advise to rephrase this sentence.

Reply: Thank you for your valuable advice. We have recharacterized this sentence to make it more specialized. The specific changes are as follows (line:83-84):

The progression of VTE is typically categorized into three distinct stages during the course of the disease.

**Comment 11.** Line 67: “a clear index” not clear what do you mean here!! Do you mean clear indication? Clarify please.

Reply: Reply: Thank you for your scientific comment. We apologize for such an ambiguous statement. What we originally intended to express in this manuscript was "indicators of detection". In conjunction with your following scientific comments, we removed the indicators of detection and unified them as therapeutic drug monitoring, and thank you for your positive suggestions on our manuscript.

**Comment 12.** Line 67 to 69: “drug treatment mechanism (hepatic and renal metabolism), variable drug half-life, treatment requirements, monitoring needs” these factors sound a bit confusing and could overlap in their meanings. Treatment mechanism, you could just say mechanism of actions. Hepatic and renal metabolism as well as half-life could all go under pharmacokinetics. Not sure what you mean by treatment requirements!!!! You could just remove it and say therapeutic drug monitoring.

Reply: Thank you for your valuable advice. We apologize for this situation. We rewrote this section to make it concise and easy to understand. The specific changes are as follows (line:89-91):

Clinical indications for each anticoagulant should be considered based on the risk of adverse drug events, Pharmacokinetics and therapeutic drug monitoring.

**Comment 13.** Line 82: “With the publication of “Good Manufacturing Practice” provide a reference here.

Reply: Thank you for your valuable advice. Here we have added a reference to “Good Manufacturing Practice for Drugs”[7].

**Reference**

[7].State Administration for Market Regulation. Good Manufacturing Practice for Drugs. 17 Jan 2011 [cited 21 Nov 2024]. Available: https://english.nmpa.gov.cn/2019-07/25/c_390613.htm

**Comment 14.** Line 85 to 92: there seems to be too much describing of DUR here. Defining what DDD or DDDC should be in the methods section and should not take this much space from the study introduction or background. This part needs rephrasing to highlight the main gaps in the literature that the study will address

Reply: Thank you for your valuable advice. We simplified the description of DUR and added the definitions of DDD and DDC to the Methods section. The specific changes are as follows (line:107-112):

The field of drug utilization research (DUR) employs a range of descriptive and analytical methods to quantitatively assess, comprehend, and evaluate the processes involved in drug prescribing, dispensing, and consumption. DUR can be utilized to identify early indicators of inappropriate drug utilization, thereby promoting the safe and effective utilization of medications and enhancing the quality of drug therapy.

**Comment 15.** Line 93 to 95: “This study aims to luzhou area through the analysis of the drug utilization research in tertiary hospital nearly five years of anticoagulant drugs usage, combining economics discussion and thinking of anticoagulant drugs selection and reasonable use.”

I advise rephrase this part and make sure its grammatically sound.

Reply: Thank you for your valuable advice. We rewrote this section to make sure the grammar is correct. In addition, we pay more attention to the content and significance of this study. The specific changes are as follows (line:113-121):

The city of Luzhou, situated in the southern region of Sichuan province, stands as a representative example with relatively high medical and economic standards within the province. Moreover, owing to factors such as advanced medical facilities and convenient transportation, tertiary-care hospitals are predominantly chosen by patients for their treatment needs. Consequently, this inclination towards tertiary-care hospitals results in a greater utilization of anticoagulant medications and facilitates a more comprehensive understanding of their usage trends. Therefore, the objective of this study is to provide an all-encompassing overview regarding the utilization and expenditure patterns of anticoagulant drugs in Luzhou's tertiary-care hospitals from 2019 to 2023 through DUR.

**Comment 16.** Overall, the methods need major structural revision. The subheading needs to be divided as follows: Data source, Data collection, then statistical analysis.

Reply: Thank you for your valuable advice. We agree with you. The journal requires that the Ethics statement section be written in the methods section. Therefore, we re-divided the subheadings: data source, data collection, statistical analysis and ethics statement.

**Comment 17.** Line 97: the subheading “Data collection” needs to be changed as I mentioned earlier and this section should be dedicated to explaining the *data sources* such as explaining the data system of the eight hospitals included in this study and if there is any coding system used to extract the necessary data.

Reply: Thank you very much for your valuable comments. We also agree with you. We have explained the part of data collection separately.

**Comment 18.** Line 99: “data collection began in March 2024”, this does not sound correct. If it’s a retrospective study and the study observational window is from 2019 to 2023.

Reply: Thank you for your valuable advice and we agree with you. We have rewritten the data sources section with the following changes (line:124-125):

This study was a retrospective multicenter study in Luzhou City, and this study's observation period spanned from 2019 to 2023.

**Comment 19.** Line 100 to 101: “Third-Level Grade A (A, B) and Third-Level Grade B (C, D, E, F, G, H)” explain to the reader what grading this is and provide references.

Reply: Thank you for your scientific comment. We supplemented the grading criteria of these hospitals and provided references. The specific changes are as follows (line:125-127):

The 8 hospitals were categorized into Grade A (A, B) and Grade B (C, D, E, F, G, H) based on the Implementation rules of tertiary hospital accreditation standards[8].

**Reference**

[8].National Health Commission of the People’s Republic of China [Internet]. Implementation rules of tertiary hospital accreditation standards (2020 edition). 2021 Oct [cited 2024 Nov 21]. Available from: http://www.nhc.gov.cn/yzygj/s7657/202110/b9fceda937184f259ecae7ece8522d24.shtml

**Comment 20.** Line 101 to 106: all this part needs to be transferred to the data collection section.

Reply: We agree with you. We have changed this section to data collection. Thank you for your positive suggestions on our manuscript.

**Comment 21.** Line 102: “medication specifications” what does this mean exactly?

Reply: Thank you for your valuable advice. "medication specifications" are intended to express specific quality standards for finished products or raw materials used. For example, the weight or amount of the main drug contained in each branch, tablet, or other unit of the preparation. We changed it to "strength" according to the FDA instructions[9].

**Reference**

[9].U.S. Food and Drug Administration [Internet]. Drugs@FDA: FDA-Approved Drugs. [cited 2024 Nov 25]. Available from: https://www.accessdata.fda.gov/scripts/cder/daf/index.cfm

**Comment 22.** Line 103: “quantities and amounts” clarify the difference.

Reply: Thank you for your valuable advice. "quantities" and "amounts" mean the same thing. We apologize for this mistake. We have modified this.

**Comment 23.** Line 103: why saying “etc.”? I believed there are five approved DOACs its better to mention all of them.

Reply: We agree with you. We have listed all the anticoagulants involved in the manuscript. Thank you for your positive suggestions on our manuscript.

**Comment 24.** Line 105: same as previous comment

Reply: We agree with you. We have listed all the anticoagulants involved in the manuscript. Thank you for your positive suggestions on our manuscript.

**Comment 25.** Line 110: here its better to change the subheading to *Data collection* and add the description of the WHO definition of DDD and DDDC.

Reply: Thank you very much for your valuable advice. We have added this section to the data collection and added a description of DDD and DDC. The specific changes are as follows (line:142-152):

The expenditure was determined using the Chinese yuan (CNY) to determine the expenditure of anticoagulants, and 7.27 CNY was equal to 1 US dollar. DDD is the assumed average maintenance dose per day of a drug used for its main indication in adults[10]. Defined daily doses (DDDs) were calculated according to DDD values provided by WHO[11]. DDDs = annual consumption of a drug/DDD value of the drug. The DDDs are primarily employed for drug utilization assessment. A higher value of DDDs indicates a greater frequency of drug use, thereby reflecting a clinical inclination towards the choice of the drug. The defined daily cost (DDC) was utilized as a measure to accurately depict the financial implications and economic burden associated with each medication[12]. DDC = an annual consumption amount of the drug/drug DDDs value. A higher DDC value indicates a more substantial economic burden on patients.

**Reference**

[10].World Health Organization [Internet]. Defined Daily Dose (DDD). [cited 2024 Nov 21]. Available from: https://www.who.int/tools/atc-ddd-toolkit/about-ddd

[11].Norwegian Institute of Public Health [Internet]. ATCDDD - ATC/DDD Index. [cited 2024 Nov 25]. Available from: https://atcddd.fhi.no/atc_ddd_index/

[12].Zhu H, Zhu J, Zhou Y, Shan L, Li C, Cui Y, et al. Impact of the National Reimbursement Drug List Negotiation Policy on Accessibility of Anticancer Drugs in China: An Interrupted Time Series Study. Front Public Health. 2022;10: 921093. doi:10.3389/fpubh.2022.921093

**Comment 26.** Line 111: create a new subheading for the statistical analysis and describe in detail how you summarized the data using descriptive statistics. Therefore remove “descriptive statistics” from this part.

Reply: We agree with you. We created a new title for the statistical analysis and described the statistical methods we used. The specific changes are as follows (line:158-163):

The statistical indicators of eight hospitals in Luzhou were calculated using Excel software. For substances with different specifications and manufacturers, the quantities were standardized through unit conversion or summation to obtain the total amount of the drug. Subsequently, Graphpad Prism 8.0 software was used to produce graphs. The trends in anticoagulants use were analyzed using linear regression analysis in SPSS 24.0, and *p* values less than 0.05 were considered statistically significant.

**Comment 27.** Line 120 to 121: “according to the management scheme suggested in the manufacturer's instructions approved by the Food and Drug Administration” not clear its better to provide an example.

Reply: Thank you for your scientific comment. Since all DDD values in the manuscript are available for query through WHO and do not require a query specification, we have modified the description in this section. The specific changes are as follows (lines 144-146):

Defined daily doses (DDDs) were calculated according to DDD values provided by WHO (For example, the DDD value for warfarin is 7.5mg).

**Comment 28.** Liner 129: “China expert consensus of warfarin anticoagulant therapy”, "Guidelines on oral anticoagulation with warfarin – fourth edition" provide reference

Reply: Thank you very much for your advice. "China expert consensus of warfarin anticoagulant therapy"[13] and "Guidelines on oral anticoagulation with warfarin - fourth edition"[14] adds a reference.

**Reference**

[13].Chinese Society of Cardiology, Committee of Cardio-cerebral-Vascular of GSC [Internet]. China expert consensus of warfarin anticoagulant therapy. 2013 [cited 2024 Nov 21]. Available from: https://cmab.yiigle.com/uploads/guide_html/%E5%8D%8E%E6%B3%95%E6%9E%97%E6%8A%97%E5%87%9D%E6%B2%BB%E7%96%97%E7%9A%84%E4%B8%AD%E5%9B%BD%E4%B8%93%E5%AE%B6%E5%85%B1%E8%AF%86.html

[14].Keeling D, Baglin T, Tait C, Watson H, Perry D, Baglin C, et al. Guidelines on oral anticoagulation with warfarin – fourth edition. Br J Haematol. 2011;154: 311–324. doi:10.1111/j.1365-2141.2011.08753.x

**Comment 29.** Line 132 to 135: provide here a proper statistical analysis section and describe in details the summary measures used in describing the results.

Reply: We agree with you. We created a new title for the statistical analysis and described the statistical methods we used. The specific changes are as follows (line:158-163):

The statistical indicators of eight hospitals in Luzhou were calculated using Excel software. For substances with different specifications and manufacturers, the quantities were standardized through unit conversion or summation to obtain the total amount of the drug. Subsequently, Graphpad Prism 8.0 software was used to produce graphs. The trends in anticoagulants use were analyzed using linear regression analysis in SPSS 24.0, and *p* values less than 0.05 were considered statistically significant.

**Comment 30.** Same as in methods the subheadings used needs modification “Different hospital drug use” is not an appropriate nor “Overall trends in anticoagulants” or “The difference of anticoagulant drugs outpatient and inpatient”. It might be better to change these subheadings to “Utilization and Trends of Anticoagulant Drugs” another for “Expenditure of Anticoagulant Drug Utilization” and another one for “Outpatient vs. Inpatient Utilization of Anticoagulant” then adjust the text accordingly.

Reply: Thank you for your valuable advice and we agree with you. We changed the subheadings of the results section to Utilization and Trends of Anticoagulants, Expenditure of Anticoagulants Utilization, and Outpatient vs Inpatient Utilization of Anticoagulants and DDC and Trends of Anticoagulants.

**Comment 31.** Line 138 to 147: this whole paragraph needs rewriting to provide a numeric description of the main results around the utilization trends with reference to Figure 1.

Figure 1 it’s not clear these results are for which year or if its for the whole observation period. Clarify please.

Reply: Thank you for your invaluable advice. We have made modifications to this section. Firstly, we have provided a detailed description of the variations in anticoagulant types utilized by different hospitals and highlighted the common characteristics observed among all eight hospitals (the top anticoagulants were consistent in terms of DDDs). Additionally, employing statistical analysis, we have identified the trending Utilization trend of the four most frequently prescribed anticoagulants in each hospital. In addition, we have also added a description of Figure 1 to prevent ambiguity. The specific changes are as follows (line:170-178):

The current landscape of anticoagulants encompasses various categories, including parenteral anticoagulants, VKA, and DOACs. In recent years (2019-2023), the utilization of anticoagulants in Luzhou eight hospitals reveals (Fig 1) that the predominant anticoagulants employed are warfarin, rivaroxaban, heparin sodium, and LMWH. For Hospital A, the top four DDDs were LMWH (1605935), warfarin (1236290), rivaroxaban (911944), and heparin sodium (86410). While other anticoagulants are being used, it can be observed that Third-Level Grade A hospitals prescribing various medications exceeds that of Third-Level Grade B hospitals. Considering the different types of drugs used in different hospitals, four anticoagulants, warfarin, rivaroxaban, heparin sodium and LMWH, which were used in eight hospitals were selected as the subjects of follow-up study.

**Comment 32.** Line 149 to 150: “The prevalence of VTE patients has been on the rise, consequently elevating the significance of anticoagulant medications” this statement is not supposed to be included here. If you think that the rise in anticoagulants utilization is related to the rise in the prevalence of VTE, you should address this in the discussion providing the appropriate references to support your claims. However, since you have not estimated the prevalence of VTE in this study then it should not be included in the results.

Reply: Reply: Thank you for your valuable advice, we also agree with your point of view. We removed this sentence to give greater prominence to the results found in this study. Thank you very much for your advice.

**Comment 33.** Line 175: “In the past five years, there has been a noticeable upward trend in the number of outpatient visits and inpatient” is something you have measured in this study (number of outpatient visits)?!!! Clarify please. If not, then just simply say between the year 20xx and 20xx ………and described anticoagulants utilization in the outpatient settings.

Reply: Thank you for your scientific comment. The number of patients with VTE was not examined in this study. We therefore deleted this sentence and simply described the use of anticoagulants in the outpatient setting.

**Comment 34.** Line 182 to 183: “This finding also indicates the prevailing utilization of oral anticoagulants as the primary choice for outpatient anticoagulation therapy” this more suitable to mention in discussion.

Reply: Thank you for your valuable advice. We also agree with you and have added this section to the discussion. In the results, more emphasis is placed on the presentation of the results of our study.

**Comment 35.** Line 187: “significant margin” you should be careful when you use the word significant as its commonly used to describe any significant statistical differences interpreted from P values or confidence intervales. Since no statistical tests were performed in this study and was only done using descriptive statistics, then its not appropriate to use this phrase.

Reply: Thank you for your scientific comment. We agree with you. This study adds inferential statistical methods to the previous study, so the presence of the term "significant (ly)" in the revised manuscript is justified.

**Comment 36.** Line 197 to 202: this whole paragraph is not suitable for results. Better to remove or include in discussion.

Reply: Thank you for your scientific comment. We added this section to the discussion. Thank you very much for your positive suggestions on our manuscript.

**Comment 37.** Table 1: it’s not clear why this table is only for warfarin? and which year is it describing? It would be clearer to include all anticoagulants and describe each year separately (for both out- and inpatient utilization).

Reply: Thank you for your valuable advice. We apologize for the ambiguities in Table 1 and have amended accordingly. This article focuses only on the indication for warfarin for several reasons. Firstly, this study aimed to understand the trend of anticoagulant use in tertiary hospitals in Luzhou city. To avoid monotonicity introduced by a simple cross-sectional study design, we included a portion of the rational use study. Secondly, due to the lack of time, we chose warfarin, the most classical and most used anticoagulant drug, to preliminarily explore its rational use. Our team is committed to further explore and improve the application of anticoagulant drugs in Luzhou City; Therefore, we expect more comprehensive findings in the near future.

**Comment 38.** The first paragraph in the discussion needs to highlight the design and purpose of this study and description in words of the main findings.

Reply: Thank you very much for your scientific comment. We have revised this section to place greater emphasis on the design, purpose, and main findings of this study. The specific changes are as follows (line:249-259):

The findings of this study present the most up-to-date data on the utilization and expenditure patterns of anticoagulants in tertiary-care hospitals within the Luzhou region (2019-2023). In recent years, VTE represents a healthcare concern with potentially grave implications, and the occurrence of acute VTE can lead to fatal outcomes. Hospitalized surgical or medical patients are particularly susceptible to VTE, and this issue persists even after discharge from the hospital. In China, the hospitalization crude rate of VTE was 2.9 in 2007 to 15.8 in 2016 per 100,000 population, with a relative increase of five-fold (*p* < 0.001). Therefore, the utilization of anticoagulants is of utmost importance. The findings from this study demonstrate a notable increase in the utilization of rivaroxaban and LMWH over the past 5 years, accompanied by a decrease in expenditure due to changes in DDC. Simultaneously, their exceptional safety profile and high efficacy distinguish them as superior options among various anticoagulants.

**Comment 39.** Line 227 to 235: I advise removing this part and link line 224 to 226 with the paragraph in line 236.

Reply: Thank you very much for your scientific comment. We also agree with you and delete this part. Thank you for your valuable advice.

**Comment 40.** Line 236 to 240: need a bit of clarifying so the reading know that you are referring to your study findings.

Reply: Thank you for your valuable advice. We apologize for our carelessness. We made our findings explicit in the discussion to make the article more explicit to the reader.

**Comment 41.** Line 257 to Line 259: again, clarify that you are referring to your study findings. Discussion is not supposed to include any reference for figures or tables.

Reply: Thank you for your valuable advice. Again, we apologize for our carelessness. We clarified our findings in the discussion and omitted references to figures or tables.

**Comment 42.** Line 270 and 290: these two paragraphs need to be linked to the findings of your study.

Reply: Thank you for your scientific comment. We modified these two paragraphs, and the results of this study were introduced on the basis of the original. Thank you very much for your valuable advice.

**Comment 43.** Line 312: “This is the reason” In observational studies it’s better to avoid definitive statements, particularly in a descriptive study like this one as you have not study any associations and you are only describing what is happening in real life settings

Reply: Thank you very much for your scientific comment. We apologize for this situation. We have removed "This is the reason" and changed the expression. The specific changes are as follows (line:333-335):

Therefore, in this study, the DDDs of rivaroxaban and LMWH exhibited an upward trend over the past five years; however, there was a decline in the expenditure and DDC of rivaroxaban and LMWH in 2021 and 2023 respectively.

**Comment 44.** Line 328 to 331: “Furthermore, our assessment solely focused on the appropriateness of warfarin prescription for its specific indication, without considering the suitability of prescribing alternative anticoagulants or the practices followed by other medical institutions in terms of anticoagulant prescriptions”. I don’t believe you have studied the appropriateness of warfarin prescribing, rather you have only mentioned the indications. Which is not enough to assess the appropriateness of prescribing, that requires extracting clinical data at the patient-level.

Reply: Thank you very much for your scientific comment. As mentioned above (Comment 37), we studied only the rational use of warfarin for various reasons. Thank you very much for your valuable comments.

**Comment 45.** Line 338 to 339: “owing to their minimal adverse reactions and remarkable therapeutic efficacy” again careful with definitive statements.

Reply: Thank you very much for your valuable advice. We modified it so that the conclusions highlight only the results of this study. The specific changes are as follows (lines 356-360):

This study shows an increase in anticoagulants utilization and spending from 2019 to 2023. In recent years, the utilization of rivaroxaban and LMWH has been increasing, and they have become the main options in this field. At the same time, the implementation of centralized procurement policy has significantly reduced the price of anticoagulants such as rivaroxaban, thus reducing the economic burden of patients.

**Reviewer #3** **Comments to the Author:** 1. Study aim statement - "This study aims to luzhou area through the analysis of the drug utilization research in tertiary hospital nearly five years of anticoagulant drugs usage, combining economics discussion and thinking of anticoagulant drugs selection and reasonable use." Need to rephrase the aim clearly and precisely.
2. Ethics statement - " The data used in this study were completely anonymized, did not require review and approval
by ethics committees, and the requirement for informed consent was waived" - For retrospective analysis of drug utilization studies, ethical approval may still be needed despite the observational nature of the study. Hence it would be appropriate you declare the proof of waiver status, as confirmed by an ethics board.
3. Figures (ALL) - Please specify the X axis and also Y axis clearly. Also add a brief description on what the figure depicts? (Example: Figure 1: Usage of anticoagulant drugs at eight hospitals and DDDs: defined daily doses)

Reply: Thank you very much for your scientific comment. For the abstract section, we significantly revised it with input from several reviewers. So that the abstract part can well represent the content of this study. In addition, for the ethical statement section, the study did not require ethical review after we asked the ethics committee and received confirmation. Finally, we apologize for the ambiguity of all figures. We added more explicit definitions for the x-and y-axes of the graphs and a short description for each graph. Thank you for your positive suggestions on our manuscript

**Comment 1.** Line 23: “2019 to 2023”. January 2019 to December 2023.

Reply: Thank you very much for your valuable advice. We agree with your point and have revised it.

**Comment 2.** Line 93-95: rephrase the aim clearly.

Reply: Thank you for your valuable advice. We redefined the objectives of the study with the following modifications (lines 113-121):

The city of Luzhou, situated in the southern region of Sichuan province, stands as a representative example with relatively high medical and economic standards within the province. Moreover, owing to factors such as advanced medical facilities and convenient transportation, tertiary-care hospitals are predominantly chosen by patients for their treatment needs. Consequently, this inclination towards tertiary-care hospitals results in a greater utilization of anticoagulant medications and facilitates a more comprehensive understanding of their usage trends. Therefore, the objective of this study is to provide an all-encompassing overview regarding the utilization and expenditure patterns of anticoagulant drugs in Luzhou's tertiary-care hospitals from 2019 to 2023 through DUR.

**Comment 3.** Line 108-109: For retrospective analysis of drug utilization studies, ethical approval may still be needed despite the observational nature of the study. Hence it would be appropriate you declare the proof of waiver status, as confirmed by an ethics board.

Reply: Thank you very much for your scientific comment. We re-stated the ethical review section with the specific modifications as follows (line:165-167):

The data used in this study were completely anonymized, confirmed by the ethics committee, did not require review and approval by the ethics committee, and the requirement for informed consent was waived.

**Comment 4.** Figure 1: Specify what is X axis for all figures (a, b, c & d), Is it the 8 hospitals? and also Y axis (Is it DDDs in CNY?

Also add a brief description on what the figure depicts? (Example: Figure 1: Usage of anticoagulant drugs at eight hospitals and DDDs: defined daily doses.

Reply: Thank you very much for your scientific comment. We redefined the X axis and Y axis of Figure 1 and added a simple description of the picture in the main text. The changes are as follows:

**Fig 1. Utilization of anticoagulants at eight hospitals from 2019 to 2023.** DDDs: defined daily doses; LMWH: low molecular weight heparin.

**Comment 5.** Figure 2: Specify what is X axis for all figures (a, b, c & d), Is it the 8 hospitals? and also Y axis (Is it DDDs in CNY?

Also add a brief description on what the figure depicts? (Example: Figure 1: Usage of anticoagulant drugs at eight hospitals and DDDs: defined daily doses.

Reply: Thank you very much for your scientific comment. Similarly, we redefined the x and y axes of Figure 2 and the following pictures and added a simple description of the pictures in the main text. The changes are as follows:

**Fig 2. Utilization of anticoagulants from 2019 to 2023. A: warfarin.** B: rivaroxaban; C: heparin sodium; D: LMWH. DDDs: defined daily doses; LMWH: low molecular weight heparin.

**Fig 3. The expenditure on anticoagulants in the period of 2019-2023(million CNY).** A: Warfarin; B: Rivaroxaban; C: Heparin sodium; D: LMWH. LMWH: low molecular weight heparin.

**Fig 4. Outpatient utilization of anticoagulants from 2019 to 2023.** A: Warfarin; B: Rivaroxaban; C: Heparin sodium; D: LMWH. DDDs: defined daily doses; LMWH: low molecular weight heparin.

**Fig 5. Inpatient utilization of anticoagulants from 2019 to 2023.** A: Warfarin; B: rivaroxaban; C: Heparin sodium; D: LMWH. DDDs: defined daily doses; LMWH: low molecular weight heparin.

**Fig 6. DDC for four Anticoagulants from 2019 to 2023.** DDC: defined daily dose cost.

**Reviewer #4** **Comments to the Author:** Title: The title is concise and captures the manuscript appropriately.
Abstract: This was well-written and sound.
Methods: The methods does not quite capture a particular study design. The authors state that it was a retrospective multicenter study. As to whether it was retrospective cross-sectional or cohort.
Results:This was well written and captures the objectives of the study.
Conclusion:The conclusion is tied in with the initial study objectives.

Reply: Your scientific comments and approval of our study are greatly appreciated. In the methods section, we added inferential statistical analysis to the original statistical analysis to make the results of this paper more convincing. In addition, this study was a retrospective cross-sectional study to objectively show the use of anticoagulants in tertiary-care hospitals in Luzhou from 2019 to 2023. Thank you again for your positive comments on our manuscript.
